# Identification of the Key Genes and Pathways in Esophageal Carcinoma

**DOI:** 10.1155/2016/2968106

**Published:** 2016-10-12

**Authors:** Peng Su, Shiwang Wen, Yuefeng Zhang, Yong Li, Yanzhao Xu, Yonggang Zhu, Huilai Lv, Fan Zhang, Mingbo Wang, Ziqiang Tian

**Affiliations:** Department of Thoracic Surgery, The Fourth Hospital of Hebei Medical University, Shijiazhuang 050011, China

## Abstract

*Objective*. Esophageal carcinoma (EC) is a frequently common malignancy of gastrointestinal cancer in the world. This study aims to screen key genes and pathways in EC and elucidate the mechanism of it.* Methods*. 5 microarray datasets of EC were downloaded from Gene Expression Omnibus. Differentially expressed genes (DEGs) were screened by bioinformatics analysis. Gene Ontology (GO) enrichment, Kyoto Encyclopedia of Genes and Genomes (KEGG) enrichment, and protein-protein interaction (PPI) network construction were performed to obtain the biological roles of DEGs in EC. Quantitative real-time polymerase chain reaction (qRT-PCR) was used to verify the expression level of DEGs in EC.* Results*. A total of 1955 genes were filtered as DEGs in EC. The upregulated genes were significantly enriched in cell cycle and the downregulated genes significantly enriched in Endocytosis. PPI network displayed CDK4 and CCT3 were hub proteins in the network. The expression level of 8 dysregulated DEGs including CDK4, CCT3, THSD4, SIM2, MYBL2, CENPF, CDCA3, and CDKN3 was validated in EC compared to adjacent nontumor tissues and the results were matched with the microarray analysis.* Conclusion*. The significantly DEGs including CDK4, CCT3, THSD4, and SIM2 may play key roles in tumorigenesis and development of EC involved in cell cycle and Endocytosis.

## 1. Introduction

Esophageal carcinoma (EC) is the sixth leading cause of cancer mortality in males and the ninth leading cause of cancer mortality in females in 2012 worldwide [1]. The highest incident rates of EC are found in Eastern Asia, Southern Africa, and Eastern Africa and the lowest incidence rate of EC is found in Western Africa [1]. Esophageal carcinoma is usually 3 to 4 times more common among men than women. The 5-year overall survival ranges from 15% to 25% [2]. In China, it is predicted that EC is the fourth leading cause of cancer deaths in males and females after lung and bronchus, stomach, and liver in 2015 [3].

EC is classified as esophageal squamous cell carcinoma (ESCC) and esophageal adenocarcinoma (EAC) according to histological type and ESCC is the predominant histological type of EC in the world [2]. It is reported that tobacco consumption, alcohol consumption, and low intake of fruits and vegetables are major risk factors for ESCC [4]. Overweight, obesity, gastroesophagus reflux disease (GERD), and Barrett's esophagus increase incidence risk of EAC [1, 5].

In addition to the above-mentioned environmental factors, abnormal expression of miRNA and genes and methylation of genes and SNPs are associated with EC tumorigenesis and development. miR-219-1 rs107822G > A polymorphism might significantly decrease ESCC risk through changing individual susceptibility to Chinese Kazakhs [5]. The cases carrying the GG variant homozygote have a significant 2.81-fold increased risk of EC [6]. miR-330-3p promotes cell growth, cell migration, and invasion and inhibits cisplatin-induced apoptosis in ESCC cells via suppression of PDCD4 expression [7]. miR-199a-5p downregulation contributes to enhancing EC cell proliferation through upregulation of mitogen-activated protein kinase kinase kinase-11 [8]. DACT2 is frequently methylated in human esophageal cancer; methylated DATC2 accelerates esophageal cancer development by activating Wnt signaling [9]. RUNX3 methylation is associated with an increased risk, progression, and poor survival in EC [10].

Currently, the molecular mechanism of EC was unclear. In this study, we used bioinformatics methods to analyze the mRNA expression data of EC, which were available on the GEO database, to identify key genes and pathways in EC, aiming to provide valuable information for further pathogenesis mechanism elucidation and provide ground work for therapeutic targets identification for EC.

## 2. Materials and Methods

### 2.1. Expression Profile Microarray

Gene expression profiles data were downloaded from the Gene Expression Omnibus (GEO) data repository (http://www.ncbi.nlm.nih.gov/geo/). The datasets of patients receiving preoperative treatment before oesophagectomy and cell lines receiving drug stimulus were excluded. Total of 5 mRNA expression datasets of EC tissues/cell lines comprising GSE53625, GSE33810, GSE17351, GSE9982, and GSE12737 were included in our study.

### 2.2. Identification of DEGs

The raw data of the mRNA expression profiles were downloaded and analyzed by R language software [11]. Background correction, quartile data normalization, and probe summarization were applied for the original data. The limma [12] method in Bioconductor (http://www.bioconductor.org/) was used to identify genes which were differentially expressed between EC and normal controls; the significance of DEGs was calculated by* t*-test and was represented by *p* value. To reduce the risk of false positives, *p* values were adjusted for multiple testing using the Benjamini-Hochberg False Discovery Rate (FDR) method. The corrected *p* value was represented by FDR [13]. FDR < 0.05 were considered as the cutoff values for DEG screening.

### 2.3. Gene Ontology Analysis

GO is a useful tool for collecting a large number of gene annotation terms [14]. The Database for Annotation, Visualization, and Integrated Discovery (DAVID) [15], is bioinformatics resources consisting of an integrated biological knowledgebase and analytic tools aimed at systematically extracting biological functional annotation from large gene/protein lists, such as being derived from high-throughput genomic experiments. To gain the in-depth understanding of the biological functions of DEGs, DAVID tool was used to obtain the enriched GO terms of DEGs based on the hypergeometric distribution to compute *p* values, which were corrected by the Benjamini and Hochberg FDR method for multiple hypothesis testing. FDR < 0.05 was set as the threshold value.

### 2.4. KEGG Enrichment Pathways

KEGG is a database resource for understanding functions of genes list from molecular level [16]. GeneCoDis3 is a valuable tool to functionally interpret results from experimental techniques in genomics [17]. This web-based application integrates different sources of information for finding groups of genes with similar biological meaning. The enrichment analysis of GeneCoDis3 is essential in the interpretation of high-throughput experiments. In the study, GeneCoDis3 software was used to test the statistical enrichment of DEGs in KEGG pathways. *p* < 0.05 was set as the threshold value.

### 2.5. PPI Interaction Network

The Biological General Repository for Interaction Datasets (BioGRID: http://thebiogrid.org/) is an open access archive of genetic and protein interactions that are curated from the primary biomedical literature for all major model organism species including budding yeast* Saccharomyces cerevisiae*, the fission yeast* Schizosaccharomyces pombe*, and the model plant* Arabidopsis thaliana*. In a word, BioGRID is a depository for genetic and protein interactions based on experimental verification [18]. The top 10 upregulated genes and top 10 downregulated genes between EC and normal controls were subjected to BioGRID database to get the predicted PPIs of these DEGs. The PPIs were visualized in Cytoscape [17].

### 2.6. qRT-PCR Validation

Total RNA of fresh paired EC tumor and adjacent nontumor specimens were extracted using TRIzol reagent (Invitrogen, CA, USA). The SuperScript III Reverse Transcription Kit (Invitrogen, CA, USA) was used to synthesize the cDNA. qRT-PCR reactions were performed using Power SYBR Green PCR Master Mix (Applied Biosystems, Foster City, CA) on the Applied Biosystems 7500 (Foster City, CA, USA). *β*-actin was used as internal control for mRNA detected. The relative expression of genes was calculated using the comparative Ct methods [19]. The PCR primers were used as shown in supplementary Table S3 in Supplementary Material available online at http://dx.doi.org/10.1155/2016/2968106.

## 3. Results

### 3.1. Identification of DEGs

Five mRNA expression profiles including 208 EC samples and 195 normal controls were downloaded and analyzed, as shown in Table 1. 208 EC samples comprised 207 squamous cell carcinoma samples and 1 adenocarcinoma sample. 1955 DEGs were identified in EC compared to normal control, including 919 upregulated and 1036 downregulated genes. The top 10 significantly upregulated and downregulated genes were listed in Table 2. The most significantly up- and downregulated genes were CDK4 and THSD4, respectively. The full list of DEGs in EC was shown in supplementary Table S1.

### 3.2. GO Analysis of DEGs

Following GO analyses for up- and downregulated DEGs, significant GO terms including biological process, cellular component, and molecular function were collected. For upregulated DEGs, cell cycle was the most significant enrichment of biological process; membrane-enclosed lumen was the highest enrichment of cellular component; nucleotide binding was the highest enrichment of molecular function, as shown in Table 3. For downregulated DEGs, response to wounding was the most significant enrichment of biological process; actin cytoskeleton was the highest enrichment of cellular component and cytoskeletal protein binding was the highest enrichment of molecular function, as shown in Table 4.

### 3.3. KEGG Enrichment Pathways of DEGs

Following KEGG enrichment analysis for DEGs, significant KEGG terms were collected. The pathways enriched by 919 upregulated DEGs were mainly related to cell cycle, RNA transport, and p53 signaling pathway (Table 5). 1036 downregulated DEGs were significantly enriched in Endocytosis, focal adhesion, and vascular smooth muscle contraction, as shown in Table 6.

### 3.4. PPI Network Construction

**Figure 1:**
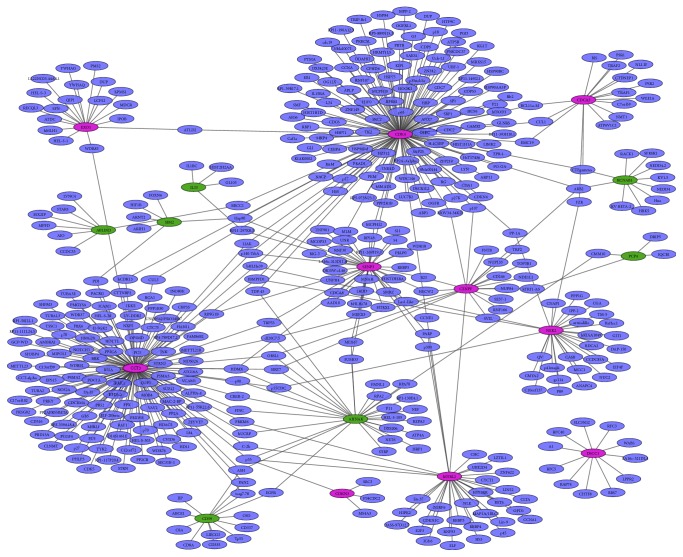
The protein-protein network of top 10 up- and downregulated DEGs in EC. The green circular nodes represent downregulation DEGs in EC; the red circular nodes represent downregulation DEGs in EC. Solid lines indicate interaction between DEGs and proteins.

Based on data from the BioGRID database, the PPI network was the top 10 upregulated and downregulated DEGs which were constructed by Cytoscape software (Figure 1). The network consisted of 451 nodes and 499 edges. In the PPI networks the nodes with high degree are defined as hub proteins. The most significant hub proteins in the PPI network were CDK4 (degree = 132) and CCT3 (degree = 127); as shown in Figure 1, the red circular nodes represent upregulated DEGs and green circular nodes represent downregulated DEGs, respectively.

### 3.5. qRT-PCR Validation of DEGs in EC Tissues

**Figure 2:**
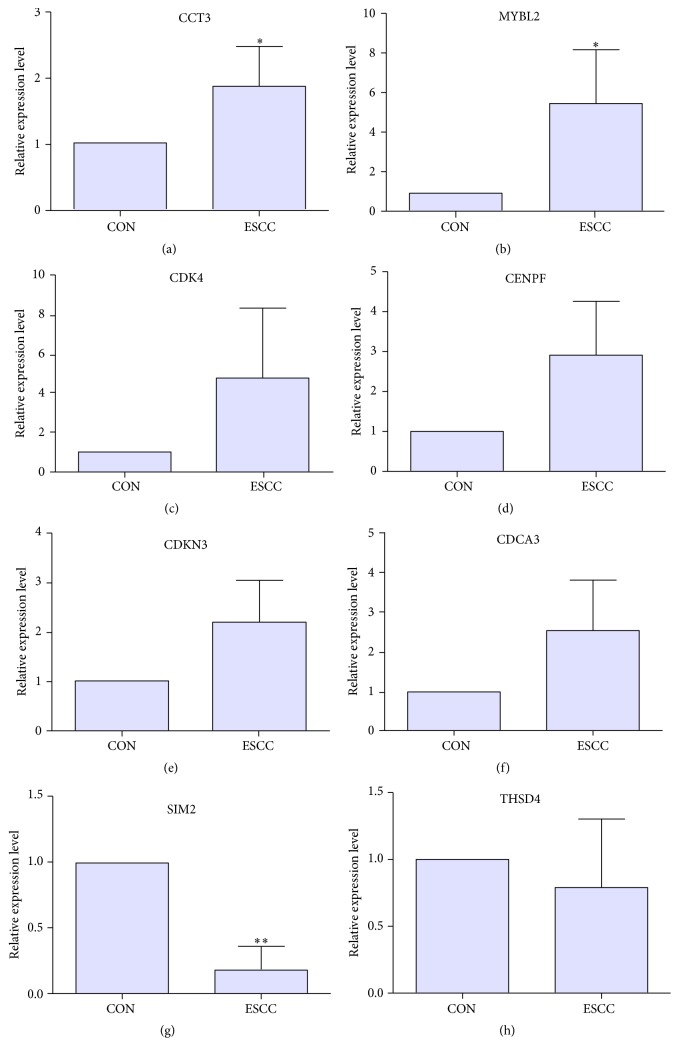
The qRT-PCR validation of the expression level of DEGs in EC compared to adjacent nontumor tissues. (a) CCT3; (b) MYBL2; (c) CDK4; (d) CENPF; (e) CDKN3; (f) CDCA3; (g) SIM2; (h) THSD4. EC: esophageal carcinoma; CON: adjacent nontumor tissues of ESCC. At least three independent experiments were performed for statistical evaluation. qRT-PCR experimental data were expressed as means ± SD. The statistical significance was evaluated using Student's* t*-test and *p* < 0.05 was considered as a significant difference.

To validate the microarray analysis data, the expression of DEGs including CCT3, CDK4, MYBL2, CENPF, CDKN3, CDCA3, THSD4, and SIM2 was detected by qRT-PCR in 5 paired EC tumor and adjacent nontumor tissues. The 5 patients received surgery treatment in Fourth Hospital of Hebei Medical University. The histological type of 5 subjects was ESCC and the detailed information of subjects was shown in supplementary Table S2. As shown in Figures 2(a) and 2(b) the expression level of CCT3 and MYBL2 was significantly upregulated in ESCC. CDK4, CENPF, CDKN3, and CDCA3 had the upregulation tendency in ESCC (Figures 2(c)–2(f)), respectively. SIM2 was significantly downregulated in ESCC (Figure 2(g)). THSD4 had the downregulation tendency in ESCC (Figure 2(h)). The qRT-PCR results were matched with the microarray analysis.

## 4. Discussion

CDK4 was identified as the most significantly upregulated gene in our microarray analysis and it had an upregulated tendency in EC tissues through the qRT-PCR validation. CDK4 was the hub protein and interacted with 132 genes in the regulatory network. CDK4 was significantly enriched in cell cycle, measles, small cell lung cancer, and pathways in cancer. CDK4 encodes cyclin-dependent kinase 4, a member of the Ser/Thr protein kinase family, which plays an important role in cell cycle G1 phase progression and G1/S transition. In our study, CDK1, CDK6, and CDK10 showed upregulation in EC. CDK1, CDK6, and CDK4 were significantly enriched in cell cycle pathway. CDK4 is overexpression in several cancer comprising of breast cancer, pancreas cancer, clear cell renal cell carcinoma, and colorectal cancer [20–23]. Downregulation of MALAT1 (long noncoding RNA metastasis-associated lung adenocarcinoma transcript 1) inhibits breast cancer cell proliferation and cell cycle progression* in vitro* and* in vivo* through miR-124 downregulation and CDK4 upregulation [20, 24]. Overexpression of cyclin D1/CDK4 is regulated by CEACAM6 and promotes cell proliferation in human pancreatic carcinoma [21]. CDK4 and CDK6 expression are decreased by miR-1 and contribute to inhibition of cell cycle progression and metastasis in clear cell renal cell carcinoma [22].

CCT3 was the top 3 upregulation DEGs in EC (Table 2). The qRT-PCR displayed that CCT3 was significantly upregulated in EC, which was in accordance with our microarray analysis (Figure 2). CCT3 interacted with 127 genes in the PPI network (Figure 1). CCT3 encodes chaperonin containing TCP1 subunit 3, a molecular chaperone, which is a member of the chaperonin containing TCP1 complex (CCT). In our study, CCT2, CCT4, CCT5, and CCT7 were upregulated in EC compared to normal controls, respectively. CCT3 depletion suppresses cell proliferation by inducing mitotic arrest at prometaphase and apoptosis eventually in HCC* in vitro*. Clinically, overexpression of CCT3 predicts poor prognosis in hepatocellular carcinoma patients after hepatectomy [25, 26]. CCT3 is significantly associated with carboplatin resistance in ovarian cancer patients after surgery treatment [27]. The proteomic-based study shows that patients with cholangiocarcinoma (CCA) which are positive for CCT3 and CCT3 might be potential biomarker for the diagnosis of CCA [28]. To our knowledge, this is the first report about CCT3 expressed status in EC and the biological function of upregulated CCT3 in EC needs further exploration.

THSD4 was the most downregulated DGE in EC through microarray analysis. The expression level of THSD4 had no significance in EC compared to normal controls but had the downregulated tendency in EC. THSD4 encodes thrombospondin type 1 domain containing 4. The methylated status of THSD4 shows positive correlation with short survival in glioblastoma patients and hypermethylation of THSD4 indicates poor survival [29]. The expression of THSD4 is regulated by GATA3 and mediates transformation of normal cells into breast cancer through deregulation of THSD4 [30]. The role of downregulated THSD4 in EC is unclear, and the investigation needs to be carried out in the future.

SIM2 was significantly downregulated in EC (Figure 2). SIM2 encodes single-minded family bHLH transcription factor 2. SIM2-s was dysregulated in glioma, prostate cancer, breast cancer, colorectal cancer, and ESCC [31–35]. SIM2s is downregulated in human breast cancer samples and it suppresses tumor activity through decreased expression of matrix metalloprotease-3. In breast cancer, SIM2s is downregulated. It is a key regulator of mammary-ductal development. SIM2s inhibition is associated with cell invasive and EMT-like phenotype through regulating matrix metalloprotease-3 expression [34, 36] It is reported that SIM2s is downregulated in 70% ESCC tissues, which is consistent with our qRT-PCR verification [35]. SIM2 overexpression results in increase of drug- and radio-sensitivities in ESCC* in vivo* and* in vitro* and patients with high expression level of SIM2 are associated with favorable prognosis before chemotherapy [35]. It is suggested that SIM2 plays vital roles in EC onset and progression.

MYBL2, CENPF, CDKN3, and CDCA3 were upregulated in EC tissues (Figure 2).* MYBL2* is frequently amplified in gastroesophageal cancer cell lines and Barrett's adenocarcinoma [37, 38].* CENPF* is frequently amplified in region around 1q32-q41 and is overexpressed in ESCC cell line [39]. CDKN3 is upregulated in 68.0% of the epithelial ovarian cancer samples and lung adenocarcinoma patients and is correlated with poor patient survival [40, 41]. CDCA3 expression status in EC was firstly reported in our study. The molecular mechanism of MYBL2, CENPF, CDKN3, and CDCA3 in EC is needed to be explored.

## 5. Conclusions

We identified 1955 DEGs comprising 919 upregulated genes and 1036 downregulated genes in EC. DEGs including CDK4, CCT3, THSD4, and SIM2 were verified in EC tissues through qRT-PCR. CDK4 and CCT3 were hub proteins in the PPI interaction network. We found that some genes including CDK4, CCT3, THSD4, and SIM2 may play essential roles in EC through cell cycle, RNA transport, Endocytosis, and focal adhesion signaling pathways. The genes could also be considered as potential candidate biomarkers for therapeutic targets for this malignancy. Furthermore, our study would shed light on the molecular mechanism underlying tumorigenesis of EC.

## Supplementary Material

The expression level of 8 candidate genes with dysregulation in esophageal carcinoma were validated through qRT-PCR. The primers for amplification of 8 genes in qRT-PCR was shown in supplementary S3.

## Figures and Tables

**Table 1:** The information of gene expression microarrays of EC.

GEO ID	Platform	Case : control	Sample type	Country	Time	Author
GSE53625	GPL18109 CBC *Homo sapiens* lncRNA + mRNA microarray V2.0	179 : 179	Esophageal squamous cell carcinoma	China	2014	Li et al. [42]
GSE33810	GPL570 [HG-U133_Plus_2] Affymetrix Human Genome U133 Plus 2.0 Array	2 : 1	Esophageal squamous cell carcinoma	HK	2013	Chen et al. [43]
GSE17351	GPL570 [HG-U133_Plus_2] Affymetrix Human Genome U133 Plus 2.0 Array	5 : 5	Esophageal squamous cell carcinoma	USA	2009	Long et al. [44]
GSE9982	GPL1928 CodeLink Human 20K ver4.1	20 : 2	Esophageal squamous cancer	Japan	2006	Shimokuni et al. [45]
GSE12737	GPL7262 Human ORESTES NoMatch 4.8k v1.0	2 : 8	Squamous cell & adenocarcinoma	Brazil	2009	Mello et al. [46]

EC: esophageal carcinoma.

**Table 2:** The top 10 up-regulated and top 10 down-regulated DEGs in EC.

Gene ID	Gene symbol	Official full name	FDR
*Upregulated (top 10)*			
1019	CDK4	Cyclin-dependent kinase 4	0.0002252
4605	MYBL2	MYB protooncogene like 2	0.0002252
7203	CCT3	Chaperonin containing TCP1 subunit 3	0.0003378
83461	CDCA3	Cell division cycle associated 3	0.0004504
1033	CDKN3	Cyclin-dependent kinase inhibitor 3	0.0004504
1063	CENPF	Centromere protein F	0.0004729
9156	EXO1	Exonuclease 1	0.0004729
79075	DSCC1	DNA replication and sister chromatid cohesion 1	0.0005405
4751	NEK2	NIMA related kinase 2	0.0005405
*Downregulated (top 10)*			
79875	THSD4	Thrombospondin type 1 domain containing 4	0.0002252
79026	AHNAK	AHNAK nucleoprotein	0.0004729
6493	SIM2	Single-minded family bHLH transcription factor 2	0.0004729
7881	KCNAB1	Potassium voltage-gated channel subfamily A member regulatory beta subunit 1	0.0005405
90865	IL33	Interleukin 33	0.0008812
55287	TMEM40	Transmembrane protein 40	0.0008812
966	CD59	CD59 molecule	0.0015608
5121	PCP4	Purkinje cell protein 4	0.0015608
22885	ABLIM3	Actin binding LIM protein family member 3	0.0016629
3590	IL11RA	Interleukin 11 receptor subunit alpha	0.0016629

EC: esophageal carcinoma; FDR: false discovery rate.

**Table 3:** GO annotation of upregulated DEGs in EC.

GO ID	GO term	Count	*p*-value	FDR
*Biological process*				
GO:0007049	Cell cycle	152	4.10*E* − 13	7.59*E* − 10
GO:0022402	Cell cycle process	118	3.44*E* − 12	6.36*E* − 09
GO:0022403	Cell cycle phase	90	2.00*E* − 10	3.71*E* − 07
GO:0000278	Mitotic cell cycle	82	5.34*E* − 10	9.87*E* − 07
GO:0051301	Cell division	67	8.80*E* − 09	1.63*E* − 05
GO:0000279	M phase	70	6.27*E* − 08	1.16*E* − 04
GO:0000087	M phase of mitotic cell cycle	49	3.38*E* − 06	0.0062547
GO:0000280	Nuclear division	48	4.65*E* − 06	0.0086014
GO:0007067	Mitosis	48	4.65*E* − 06	0.0086014
GO:0048285	Organelle fission	49	6.41*E* − 06	0.011854
GO:0033554	Cellular response to stress	95	2.02*E* − 05	0.0373322
*Cellular component*				
GO:0031974	Membrane-enclosed lumen	276	1.12*E* − 10	1.65*E* − 07
GO:0043233	Organelle lumen	270	2.41*E* − 10	3.56*E* − 07
GO:0043232	Intracellular non-membrane-bounded organelle	359	8.74*E* − 10	1.29*E* − 06
GO:0043228	Non-membrane-bounded organelle	359	8.74*E* − 10	1.29*E* − 06
GO:0070013	Intracellular organelle lumen	259	4.54*E* − 09	6.71*E* − 06
GO:0031981	Nuclear lumen	216	1.90*E* − 08	2.80*E* − 05
GO:0000775	Chromosome, centromeric region	36	2.52*E* − 08	3.72*E* − 05
GO:0005829	Cytosol	192	1.36*E* − 06	0.0020016
GO:0015630	Microtubule cytoskeleton	92	4.62*E* − 06	0.0068255
GO:0000793	Condensed chromosome	32	6.75*E* − 06	0.009972
GO:0000779	Condensed chromosome, centromeric region	21	7.55*E* − 06	0.011151
GO:0044427	Chromosomal part	69	9.92*E* − 06	0.0146408
GO:0005635	Nuclear envelope	43	1.37*E* − 05	0.0202598
GO:0000777	Condensed chromosome kinetochore	19	1.48*E* − 05	0.0219025
GO:0005694	Chromosome	78	1.75*E* − 05	0.02589
GO:0000776	Kinetochore	22	2.72*E* − 05	0.0401619
*Molecular function*				
GO:0000166	Nucleotide binding	305	5.53*E* − 06	0.0090275
GO:0017076	Purine nucleotide binding	266	5.55*E* − 06	0.0090714
GO:0030554	Adenyl nucleotide binding	223	1.07*E* − 05	0.0175078
GO:0001883	Purine nucleoside binding	225	1.49*E* − 05	0.0242774
GO:0032555	Purine ribonucleotide binding	252	2.35*E* − 05	0.0383944
GO:0032553	Ribonucleotide binding	252	2.35*E* − 05	0.0383944
GO:0001882	Nucleoside binding	225	2.44*E* − 05	0.0398342

EC: esophageal carcinoma; FDR: false discovery rate.

**Table 4:** GO annotation of downregulated DEGs in EC.

GO ID	GO term	Count	*p* value	FDR
*Biological process*				
GO:0009611	Response to wounding	65	1.98*E* − 08	3.57*E* − 05
GO:0042060	Wound healing	33	5.75*E* − 08	1.04*E* − 04
GO:0030097	Hemopoiesis	32	1.85*E* − 05	0.0334238
GO:0007167	Enzyme linked receptor protein signaling pathway	41	2.03*E* − 05	0.0365533
GO:0030036	Actin cytoskeleton organization	31	2.05*E* − 05	0.0370181
GO:0048534	Hemopoietic or lymphoid organ development	34	2.06*E* − 05	0.0372021
GO:0007155	Cell adhesion	69	2.10*E* − 05	0.0378896
GO:0042692	Muscle cell differentiation	21	2.14*E* − 05	0.0386651
GO:0022610	Biological adhesion	69	2.19*E* − 05	0.0394751
GO:0007178	Transmembrane receptor protein serine/threonine kinase signaling pathway	19	2.53*E* − 05	0.0456886
*Cellular component*				
GO:0015629	Actin cytoskeleton	36	5.84*E* − 06	0.008305
GO:0005794	Golgi apparatus	83	7.36*E* − 06	0.0104637
GO:0005856	Cytoskeleton	118	1.23*E* − 05	0.0175254
*Molecular function*				
GO:0008092	Cytoskeletal protein binding	59	8.55*E* − 07	0.0013403

EC: esophageal carcinoma; FDR: false discovery rate.

**Table 5:** The KEGG pathway enrichment of up-regulated DEGs in EC.

KEGG ID	KEGG terms	Count	FDR	Genes
hsa04110	Cell cycle	19	7.86*E* − 08	CDK6, CCNE2, CCNB2, FZR1, CCNA2, CDC7, YWHAQ, MCM7, CCNE1, CDK4, E2F5, CCNB1, MAD2L1, CDC25B, MCM6, BUB1, RBL1, MCM2, CDK1
hsa03013	RNA transport	20	1.09*E* − 07	RAN, EIF3H, NUP43, UBE2I, NUP133, MAGOHB, POP5, THOC5, CLNS1A, NUP205, GEMIN6, NUP93, NUP62, SUMO1, EIF2S2, NUP153, RANGAP1, NUP160, RPP25, DDX20
hsa04115	p53 signaling pathway	5	2.90*E* − 06	CCNE2, CCNB2, CCNE1, CCNB1, CDK1
hsa04914	Progesterone-mediated oocyte maturation	8	1.42*E* − 05	CCNB2, FZR1, CCNA2, CCNB1, MAD2L1, CDC25B, BUB1, CDK1
hsa03050	Proteasome	9	1.56*E* − 05	PSMD7, SHFM1, PSMD3, PSMA5, PSMB1, PSMB3, PSMA3, PSMD4, PSMA7
hsa03040	Spliceosome	15	1.66*E* − 05	SNRPC, SRSF9, XAB2, MAGOHB, NAA38, BUD31, SNRPF, NHP2L1, SRSF3, PQBP1, USP39, SNRNP40, SNRPD1, SNRPD2, SF3B2
hsa03030	DNA replication	8	4.24*E* − 05	RNASEH2A, RNASEH1, MCM7, POLE2, MCM6, RNASEH2C, MCM2, RFC4
hsa03008	Ribosome biogenesis in eukaryotes	11	4.37*E* − 05	UTP18, RAN, UTP15, NOP56, DKC1, POP5, FBL, NHP2L1, TCOF1, GNL3L, RPP25
hsa03440	Homologous recombination	7	4.37*E* − 05	SHFM1, MRE11A, RAD54B, XRCC2, RAD54L, BLM, TOP3A
hsa04114	Oocyte meiosis	8	8.96*E* − 05	CCNE2, CCNB2, YWHAQ, CCNE1, CCNB1, MAD2L1, BUB1, CDK1
hsa05162	Measles	4	0.0001531	CDK6, CCNE2, CCNE1, CDK4
hsa05222	Small cell lung cancer	4	0.0001531	CDK6, CCNE2, CCNE1, CDK4
hsa05200	Pathways in cancer	23	0.0001815	VEGFB, CDK6, MTOR, FH, CCNE2, LEF1, BIRC5, CCNE1, CDK4, TCEB1, MSH6, EGF, FZD2, TFG, CKS1B, TRAF4, HSP90AA1, TRAF3, PPARG, HSP90AB1, FGF12, PIAS4, STK4
hsa00510	N-Glycan biosynthesis	8	0.000312	RFT1, ALG10, RPN2, ALG10B, ALG1, MOGS, ALG5, B4GALT2

EC: esophageal carcinoma; FDR: false discovery rate.

**Table 6:** The KEGG pathway enrichment of downregulated DEGs in EC.

KEGG ID	KEGG terms	Count	FDR	Genes
hsa04144	Endocytosis	23	5.22*E* − 06	STAMBP, RAB11FIP5, SH3KBP1, KIT, FOLR2, F2R, TGFBR2, VPS4B, SH3GLB1, CHMP5, CXCR2, PDGFRA, CLTB, FOLR1, STAM2, ARAP2, DAB2, EEA1, PDCD6IP, RAB11FIP2, CBL, EPN3, VPS37B
hsa04510	Focal adhesion	19	0.000354	ITGA1, ZYX, LAMB2, MYLK, IGF1, CCND2, ITGA2, RAP1A, PDGFRA, ITGA5, TNXB, VWF, PIK3R1, JUN, COL6A2, BCL2, ROCK1, MYL12A, THBS3
hsa04270	Vascular smooth muscle contraction	14	0.000383	JMJD7-PLA2G4B, MYLK, ADCY9, GNA13, PRKG1, ITPR2, PPP1R12B, GNAQ, MYH11, ACTG2, ROCK1, PLA2G2A, MRVI1, ITPR1
hsa00330	Arginine and proline metabolism	4	0.000425	ALDH7A1, MAOB, GATM, MAOA
hsa04360	Axon guidance	15	0.000456	EPHA1, ROBO1, SEMA4B, DPYSL2, ABLIM3, PPP3CC, NCK2, GNAI2, SEMA3F, PPP3CA, RGS3, NTN1, ROCK1, PPP3CB, EFNB2
hsa04020	Calcium signaling pathway	5	0.00046	PPP3CC, ITPR2, PPP3CA, PPP3CB, ITPR1
hsa04662	B cell receptor signaling pathway	4	0.000508	PPP3CC, JUN, PPP3CA, PPP3CB
hsa05014	Amyotrophic lateral sclerosis	3	0.000583	PPP3CC, PPP3CA, PPP3CB
hsa00340	Histidine metabolism	3	0.000583	ALDH7A1, MAOB, MAOA
hsa04720	Long-term potentiation	6	0.000623	PPP3CC, ITPR2, GNAQ, PPP3CA, PPP3CB, ITPR1
hsa04114	Oocyte meiosis	6	0.00068	ADCY9, PPP3CC, ITPR2, PPP3CA, PPP3CB, ITPR1
hsa04730	Long-term depression	10	0.000701	JMJD7-PLA2G4B, IGF1, GNA13, PRKG1, ITPR2, PPP2CB, GNAQ, GNAI2, PLA2G2A, ITPR1
hsa04141	Protein processing in endoplasmic reticulum	16	0.000709	SEC63, UBE2J1, EIF2AK3, ATF6, CRYAB, UBE2D3, DNAJB2, SEC31B, MAN1A1, ERO1L, BCL2, HERPUD1, DNAJC3, UBQLN2, RAD23B, LMAN1
hsa04912	GnRH signaling pathway	12	0.000736	JMJD7-PLA2G4B, MMP2, ADCY9, MAP3K3, HBEGF, ITPR2, MAPK7, GNAQ, MAP3K4, JUN, PLA2G2A, ITPR1
hsa00280	Valine, leucine, and isoleucine degradation	8	0.000738	ALDH7A1, ACADM, HMGCS1, MUT, ABAT, ACADSB, ACAD8, AUH

EC: esophageal cancer; FDR: false discovery rate.

## References

[1] Siegel R., Naishadham D., Jemal A. (2012). Cancer statistics, 2012. *CA: A Cancer Journal for Clinicians*.

[2] Enzinger P. C., Mayer R. J. (2003). Esophageal cancer. *The New England Journal of Medicine*.

[3] Chen W., Zheng R., Baade P. D. (2016). Cancer statistics in China, 2015. *CA: A Cancer Journal for Clinicians*.

[4] Arnal M. J. D., Arenas Á. F., Arbeloa Á. L. (2015). Esophageal cancer: risk factors, screening and endoscopic treatment in Western and Eastern countries. *World Journal of Gastroenterology*.

[5] Song X., You W., Zhu J. (2015). A genetic variant in miRNA-219-1 is associated with risk of esophageal squamous cell carcinoma in Chinese Kazakhs. *Disease Markers*.

[6] Ye B., Ji C.-Y., Zhao Y., Li W., Feng J., Zhang X. (2014). Single nucleotide polymorphism at alcohol dehydrogenase-1B is associated with risk of esophageal squamous cell carcinoma. *Cancer Cell International*.

[7] Meng H., Wang K., Chen X. (2015). MicroRNA-330-3p functions as an oncogene in human esophageal cancer by targeting programmed cell death 4. *American Journal of Cancer Research*.

[8] Byrnes K. A., Phatak P., Mansour D. (2016). Overexpression of miR-199a-5p decreases esophageal cancer cell proliferation through repression of mitogen-activated protein kinase kinase kinase-11 (MAP3K11). *Oncotarget*.

[9] Zhang M., Linghu E., Zhan Q. (2016). Methylation of *DACT2* accelerates esophageal cancer development by activating Wnt signaling. *Oncotarget*.

[10] Wang Y., Qin X., Wu J. (2014). Association of promoter methylation of RUNX3 gene with the development of esophageal cancer: a meta analysis. *PLoS ONE*.

[11] Gautier L., Cope L., Bolstad B. M., Irizarry R. A. (2004). Affy—analysis of Affymetrix GeneChip data at the probe level. *Bioinformatics*.

[12] Smyth G. K. (2005). Limma: linear models for microarray data. *Bioinformatics and Computational Biology Solutions Using R and Bioconductor*.

[13] Benjamini Y., Hochberg Y. (1995). Controlling the false discovery rate: a practical and powerful approach to multiple testing. *Journal of the Royal Statistical Society, Series B: Methodological*.

[14] Ashburner M., Ball C. A., Blake J. A. (2000). Gene ontology: tool for the unification of biology. *Nature Genetics*.

[15] Alvord W. G., Roayaei J., Stephens R. (2007). The DAVID gene functional classification tool: a novel biological module-centric algorithm to functionally analyze large gene lists. *Genome Biology*.

[16] Kanehisa M., Araki M., Goto S. (2008). KEGG for linking genomes to life and the environment. *Nucleic Acids Research*.

[17] Shannon P., Markiel A., Ozier O. (2003). Cytoscape: a software environment for integrated models of biomolecular interaction networks. *Genome Research*.

[18] Chatr-Aryamontri A., Breitkreutz B.-J., Oughtred R. (2015). The BioGRID interaction database: 2015 update. *Nucleic Acids Research*.

[19] Schmittgen T. D., Livak K. J. (2008). Analyzing real-time PCR data by the comparative CT method. *Nature Protocols*.

[20] Feng T., Shao F., Wu Q. (2016). miR-124 downregulation leads to breast cancer progression via LncRNA-MALAT1 regulation and CDK4/E2F1 signal activation. *Oncotarget*.

[21] Yan L., Wang Y., Wang Z.-Z. (2016). Cell motility and spreading promoted by CEACAM6 through cyclin D1/CDK4 in human pancreatic carcinoma. *Oncology Reports*.

[22] Xiao H., Zeng J., Li H. (2015). MiR-1 downregulation correlates with poor survival in clear cell renal cell carcinoma where it interferes with cell cycle regulation and metastasis. *Oncotarget*.

[23] Wang J., Yu S., Cui L. (2015). Role of SMC1A overexpression as a predictor of poor prognosis in late stage colorectal cancer. *BMC Cancer*.

[24] Feng T., Xu D., Tu C. (2015). miR-124 inhibits cell proliferation in breast cancer through downregulation of CDK4. *Tumor Biology*.

[25] Zhang Y., Wang Y., Wei Y. (2016). Molecular chaperone CCT3 supports proper mitotic progression and cell proliferation in hepatocellular carcinoma cells. *Cancer Letters*.

[26] Cui X., Hu Z.-P., Li Z., Gao P.-J., Zhu J.-Y. (2015). Overexpression of chaperonin containing TCP1, subunit 3 predicts poor prognosis in hepatocellular carcinoma. *World Journal of Gastroenterology*.

[27] Pénzváltó Z., Lánczky A., Lénárt J. (2014). MEK1 is associated with carboplatin resistance and is a prognostic biomarker in epithelial ovarian cancer. *BMC Cancer*.

[28] Shi Y., Deng X., Zhan Q. (2013). A prospective proteomic-based study for identifying potential biomarkers for the diagnosis of cholangiocarcinoma. *Journal of Gastrointestinal Surgery*.

[29] Ma J., Hou X., Li M. (2015). Genome-wide methylation profiling reveals new biomarkers for prognosis prediction of glioblastoma. *Journal of Cancer Research and Therapeutics*.

[30] Cohen H., Ben-Hamo R., Gidoni M. (2014). Shift in GATA3 functions, and GATA3 mutations, control progression and clinical presentation in breast cancer. *Breast Cancer Research*.

[31] Su Y., Wang J., Zhang X. (2014). Targeting SIM2-s decreases glioma cell invasion through mesenchymal–epithelial transition. *Journal of Cellular Biochemistry*.

[32] Su Y., He Q., Deng L. (2014). MiR-200a impairs glioma cell growth, migration, and invasion by targeting SIM2-s. *NeuroReport*.

[33] Xiao W. H., Qu X. L., Li X. M. (2015). Identification of commonly dysregulated genes in colorectal cancer by integrating analysis of RNA-Seq data and qRT-PCR validation. *Cancer Gene Therapy*.

[34] Laffin B., Wellberg E., Kwak H.-I. (2008). Loss of singleminded-2s in the mouse mammary gland induces an epithelial-mesenchymal transition associated with up-regulation of slug and matrix metalloprotease 2. *Molecular and Cellular Biology*.

[35] Komatsu M., Sasaki H. (2014). DNA methylation is a key factor in understanding differentiation phenotype in esophageal squamous cell carcinoma. *Epigenomics*.

[36] Kwak H.-I., Gustafson T., Metz R. P., Laffin B., Schedin P., Porter W. W. (2007). Inhibition of breast cancer growth and invasion by single-minded 2s. *Carcinogenesis*.

[37] Rosenberg C., Geelen E., IJszenga M. J. (2002). Spectrum of genetic changes in gastro-esophageal cancer cell lines determined by an integrated molecular cytogenetic approach. *Cancer Genetics and Cytogenetics*.

[38] Albrecht B., Hausmann M., Zitzelsberger H. (2004). Array-based comparative genomic hybridization for the detection of DNA sequence copy number changes in Barrett's adenocarcinoma. *The Journal of Pathology*.

[39] Komatsu S., Imoto I., Tsuda H. (2009). Overexpression of SMYD2 relates to tumor cell proliferation and malignant outcome of esophageal squamous cell carcinoma. *Carcinogenesis*.

[40] Fan C., Chen L., Huang Q. (2015). Overexpression of major CDKN3 transcripts is associated with poor survival in lung adenocarcinoma. *British Journal of Cancer*.

[41] Li T., Xue H., Guo Y., Guo K. (2014). CDKN3 is an independent prognostic factor and promotes ovarian carcinoma cell proliferation in ovarian cancer. *Oncology Reports*.

[42] Li J., Chen Z., Tian L. (2014). LncRNA profile study reveals a three-lncRNA signature associated with the survival of patients with oesophageal squamous cell carcinoma. *Gut*.

[43] Chen K., Li Y., Dai Y. (2013). Characterization of tumor suppressive function of *cornulin* in esophageal squamous cell carcinoma. *PLoS ONE*.

[44] Long A., Giroux V., Whelan K. A. (2015). WNT10A promotes an invasive and self-renewing phenotype in esophageal squamous cell carcinoma. *Carcinogenesis*.

[45] Shimokuni T., Tanimoto K., Hiyama K. (2006). Chemosensitivity prediction in esophageal squamous cell carcinoma: novel marker genes and efficacy-prediction formulae using their expression data. *International Journal of Oncology*.

[46] Mello B. P., Abrantes E. F., Torres C. H. (2009). No-match ORESTES explored as tumor markers. *Nucleic Acids Research*.

