# Supplementary material for: Identification of the Key Genes and Pathways in Esophageal Carcinoma
Source: Gastroenterol Res Pract. 2016 Oct 12;2016:2968106. doi: 10.1155/2016/2968106 (PMC5080515; doi:10.1155/2016/2968106)
Supplement: Supplementary file 1 — The expression level of 8 candidate genes with dysregulation in esophageal carcinoma were validated through qRT-PCR. The primers for amplification of 8 genes in qRT-PCR was shown in supplementary S3. [file 2968106.f1.docx]

**Table S1 The full list of DEGs in EC**

| **Gene** | ***p*-value** | **FDR** |
| --- | --- | --- |
| **Up-regulated genes** |  |  |
| CDK4 | 8.50E-08 | 0.0002252 |
| MYBL2 | 8.50E-08 | 0.0002252 |
| CCT3 | 1.70E-07 | 0.0003378 |
| CDCA3 | 3.40E-07 | 0.0004504 |
| CDKN3 | 3.40E-07 | 0.0004504 |
| CENPF | 5.95E-07 | 0.0004729 |
| EXO1 | 5.95E-07 | 0.0004729 |
| DSCC1 | 1.02E-06 | 0.0005405 |
| NEK2 | 1.02E-06 | 0.0005405 |
| SENP3 | 1.02E-06 | 0.0005405 |
| TEAD4 | 1.02E-06 | 0.0005405 |
| BUB1 | 1.61E-06 | 0.0007131 |
| DDX11 | 1.61E-06 | 0.0007131 |
| POLR2D | 1.61E-06 | 0.0007131 |
| MCM2 | 2.55E-06 | 0.0008812 |
| RFC4 | 2.55E-06 | 0.0008812 |
| UBE2T | 2.55E-06 | 0.0008812 |
| B4GALT2 | 3.82E-06 | 0.001216 |
| CDC7 | 3.82E-06 | 0.001216 |
| ASF1B | 5.69E-06 | 0.0015608 |
| SPC25 | 5.69E-06 | 0.0015608 |
| BLM | 8.16E-06 | 0.0016629 |
| CCT5 | 8.16E-06 | 0.0016629 |
| CHAF1A | 8.16E-06 | 0.0016629 |
| DSN1 | 8.16E-06 | 0.0016629 |
| FZD2 | 8.16E-06 | 0.0016629 |
| KIF15 | 8.16E-06 | 0.0016629 |
| NCAPG | 8.16E-06 | 0.0016629 |
| CENPN | 1.16E-05 | 0.0019141 |
| DEPDC1B | 1.16E-05 | 0.0019141 |
| EPB41L4B | 1.16E-05 | 0.0019141 |
| NCAPH | 1.16E-05 | 0.0019141 |
| PSMB3 | 1.16E-05 | 0.0019141 |
| TPRA1 | 1.16E-05 | 0.0019141 |
| CDK1 | 1.60E-05 | 0.002016 |
| CHKA | 1.60E-05 | 0.002016 |
| CKS1B | 1.60E-05 | 0.002016 |
| EXOSC2 | 1.60E-05 | 0.002016 |
| MSH6 | 1.60E-05 | 0.002016 |
| NDUFB9 | 1.60E-05 | 0.002016 |
| SPAG5 | 1.60E-05 | 0.002016 |
| TBRG4 | 1.60E-05 | 0.002016 |
| TGIF2 | 1.60E-05 | 0.002016 |
| TMEM97 | 1.60E-05 | 0.002016 |
| ATAD2 | 2.19E-05 | 0.0021518 |
| CBX2 | 2.19E-05 | 0.0021518 |
| CSE1L | 2.19E-05 | 0.0021518 |
| HAUS7 | 2.19E-05 | 0.0021518 |
| HOXA10 | 2.19E-05 | 0.0021518 |
| JMJD4 | 2.19E-05 | 0.0021518 |
| NUDT1 | 2.19E-05 | 0.0021518 |
| REXO4 | 2.19E-05 | 0.0021518 |
| RSRC1 | 2.19E-05 | 0.0021518 |
| TRAF3 | 2.19E-05 | 0.0021518 |
| UBE2C | 2.19E-05 | 0.0021518 |
| UNG | 2.19E-05 | 0.0021518 |
| ANKRD39 | 2.95E-05 | 0.0023679 |
| CCNE1 | 2.95E-05 | 0.0023679 |
| CDCA8 | 2.95E-05 | 0.0023679 |
| CLPB | 2.95E-05 | 0.0023679 |
| DKC1 | 2.95E-05 | 0.0023679 |
| NOP56 | 2.95E-05 | 0.0023679 |
| NUP160 | 2.95E-05 | 0.0023679 |
| PAFAH1B3 | 2.95E-05 | 0.0023679 |
| SHFM1 | 2.95E-05 | 0.0023679 |
| SLC25A15 | 2.95E-05 | 0.0023679 |
| ATG4D | 3.93E-05 | 0.002585 |
| BIRC5 | 3.93E-05 | 0.002585 |
| C8orf76 | 3.93E-05 | 0.002585 |
| CENPBD1 | 3.93E-05 | 0.002585 |
| CISD2 | 3.93E-05 | 0.002585 |
| GEMIN6 | 3.93E-05 | 0.002585 |
| MDK | 3.93E-05 | 0.002585 |
| MIIP | 3.93E-05 | 0.002585 |
| PPP5C | 3.93E-05 | 0.002585 |
| RAB3IP | 3.93E-05 | 0.002585 |
| RAD54L | 3.93E-05 | 0.002585 |
| SIVA1 | 3.93E-05 | 0.002585 |
| UTP23 | 3.93E-05 | 0.002585 |
| ACTR5 | 5.18E-05 | 0.0030476 |
| DHODH | 5.18E-05 | 0.0030476 |
| MAPK12 | 5.18E-05 | 0.0030476 |
| OGFR | 5.18E-05 | 0.0030476 |
| PFDN6 | 5.18E-05 | 0.0030476 |
| RIPK2 | 5.18E-05 | 0.0030476 |
| SLC19A1 | 5.18E-05 | 0.0030476 |
| SNRPC | 5.18E-05 | 0.0030476 |
| TMEM106C | 5.18E-05 | 0.0030476 |
| WDHD1 | 5.18E-05 | 0.0030476 |
| CENPM | 6.76E-05 | 0.0034875 |
| CEP78 | 6.76E-05 | 0.0034875 |
| CIT | 6.76E-05 | 0.0034875 |
| KIAA0101 | 6.76E-05 | 0.0034875 |
| KIAA2013 | 6.76E-05 | 0.0034875 |
| MAGOHB | 6.76E-05 | 0.0034875 |
| MCM7 | 6.76E-05 | 0.0034875 |
| NUP133 | 6.76E-05 | 0.0034875 |
| PPP1CA | 6.76E-05 | 0.0034875 |
| RAD54B | 6.76E-05 | 0.0034875 |
| SLC25A10 | 6.76E-05 | 0.0034875 |
| TOP1MT | 6.76E-05 | 0.0034875 |
| ARHGEF19 | 8.71E-05 | 0.0041714 |
| C1orf226 | 8.71E-05 | 0.0041714 |
| E2F5 | 8.71E-05 | 0.0041714 |
| KDM1A | 8.71E-05 | 0.0041714 |
| KIF22 | 8.71E-05 | 0.0041714 |
| LRRC8D | 8.71E-05 | 0.0041714 |
| MRPL28 | 8.71E-05 | 0.0041714 |
| PSMA7 | 8.71E-05 | 0.0041714 |
| UBFD1 | 8.71E-05 | 0.0041714 |
| ABHD11 | 0.0001116 | 0.0046932 |
| AIMP2 | 0.0001116 | 0.0046932 |
| ASPM | 0.0001116 | 0.0046932 |
| CENPE | 0.0001116 | 0.0046932 |
| CEP72 | 0.0001116 | 0.0046932 |
| DARS2 | 0.0001116 | 0.0046932 |
| HOXD11 | 0.0001116 | 0.0046932 |
| HSP90AA1 | 0.0001116 | 0.0046932 |
| MCM10 | 0.0001116 | 0.0046932 |
| RNF7 | 0.0001116 | 0.0046932 |
| SERPINH1 | 0.0001116 | 0.0046932 |
| STMN1 | 0.0001116 | 0.0046932 |
| TIPIN | 0.0001116 | 0.0046932 |
| WHSC1 | 0.0001116 | 0.0046932 |
| BRCA1 | 0.0001415 | 0.0054078 |
| CNIH4 | 0.0001415 | 0.0054078 |
| DCAF13 | 0.0001415 | 0.0054078 |
| FOXM1 | 0.0001415 | 0.0054078 |
| GMNN | 0.0001415 | 0.0054078 |
| GTF2H4 | 0.0001415 | 0.0054078 |
| HAUS5 | 0.0001415 | 0.0054078 |
| KIF23 | 0.0001415 | 0.0054078 |
| KPNA2 | 0.0001415 | 0.0054078 |
| MTHFD2 | 0.0001415 | 0.0054078 |
| RAN | 0.0001415 | 0.0054078 |
| TRIP13 | 0.0001415 | 0.0054078 |
| TSNAX | 0.0001415 | 0.0054078 |
| UBE2O | 0.0001415 | 0.0054078 |
| VAPB | 0.0001415 | 0.0054078 |
| CCNB1 | 0.0001784 | 0.0058354 |
| CCNB2 | 0.0001784 | 0.0058354 |
| COX17 | 0.0001784 | 0.0058354 |
| DCAF17 | 0.0001784 | 0.0058354 |
| DDX27 | 0.0001784 | 0.0058354 |
| DHFRL1 | 0.0001784 | 0.0058354 |
| EED | 0.0001784 | 0.0058354 |
| EHMT2 | 0.0001784 | 0.0058354 |
| FARSA | 0.0001784 | 0.0058354 |
| GNB1L | 0.0001784 | 0.0058354 |
| HOXD10 | 0.0001784 | 0.0058354 |
| LPCAT3 | 0.0001784 | 0.0058354 |
| MKNK1 | 0.0001784 | 0.0058354 |
| MYNN | 0.0001784 | 0.0058354 |
| POLR3D | 0.0001784 | 0.0058354 |
| PPM1G | 0.0001784 | 0.0058354 |
| SF3B2 | 0.0001784 | 0.0058354 |
| SFXN1 | 0.0001784 | 0.0058354 |
| ZIC2 | 0.0001784 | 0.0058354 |
| ALG1 | 0.000223 | 0.006331 |
| C1orf112 | 0.000223 | 0.006331 |
| CCT4 | 0.000223 | 0.006331 |
| CNOT7 | 0.000223 | 0.006331 |
| DHFR | 0.000223 | 0.006331 |
| FBRSL1 | 0.000223 | 0.006331 |
| FLOT1 | 0.000223 | 0.006331 |
| HPS3 | 0.000223 | 0.006331 |
| MAP4K2 | 0.000223 | 0.006331 |
| MCM6 | 0.000223 | 0.006331 |
| NCAPG2 | 0.000223 | 0.006331 |
| NUP93 | 0.000223 | 0.006331 |
| PARP1 | 0.000223 | 0.006331 |
| PEX13 | 0.000223 | 0.006331 |
| PYCR1 | 0.000223 | 0.006331 |
| RBM19 | 0.000223 | 0.006331 |
| RBM4B | 0.000223 | 0.006331 |
| RPP25 | 0.000223 | 0.006331 |
| SNX8 | 0.000223 | 0.006331 |
| SSB | 0.000223 | 0.006331 |
| TMEM194A | 0.000223 | 0.006331 |
| UTP18 | 0.000223 | 0.006331 |
| CCDC85C | 0.0002772 | 0.0069959 |
| CDT1 | 0.0002772 | 0.0069959 |
| CNNM4 | 0.0002772 | 0.0069959 |
| HNRNPA3P1 | 0.0002772 | 0.0069959 |
| HOXC10 | 0.0002772 | 0.0069959 |
| KTI12 | 0.0002772 | 0.0069959 |
| LIG3 | 0.0002772 | 0.0069959 |
| MAD2L1 | 0.0002772 | 0.0069959 |
| NAT14 | 0.0002772 | 0.0069959 |
| NSUN3 | 0.0002772 | 0.0069959 |
| NTHL1 | 0.0002772 | 0.0069959 |
| PMF1 | 0.0002772 | 0.0069959 |
| RBM38 | 0.0002772 | 0.0069959 |
| RNASEH1 | 0.0002772 | 0.0069959 |
| SBNO1 | 0.0002772 | 0.0069959 |
| STC2 | 0.0002772 | 0.0069959 |
| TIMM17A | 0.0002772 | 0.0069959 |
| TOP3A | 0.0002772 | 0.0069959 |
| BPNT1 | 0.0003421 | 0.0078156 |
| CIAPIN1 | 0.0003421 | 0.0078156 |
| CNPY3 | 0.0003421 | 0.0078156 |
| DHX34 | 0.0003421 | 0.0078156 |
| IGF2BP3 | 0.0003421 | 0.0078156 |
| ILF2 | 0.0003421 | 0.0078156 |
| KIAA0895 | 0.0003421 | 0.0078156 |
| MRPL21 | 0.0003421 | 0.0078156 |
| NELL2 | 0.0003421 | 0.0078156 |
| PAICS | 0.0003421 | 0.0078156 |
| PTPN2 | 0.0003421 | 0.0078156 |
| SIAH1 | 0.0003421 | 0.0078156 |
| SLC4A1AP | 0.0003421 | 0.0078156 |
| SNRNP40 | 0.0003421 | 0.0078156 |
| TCOF1 | 0.0003421 | 0.0078156 |
| VKORC1L1 | 0.0003421 | 0.0078156 |
| CHRNA5 | 0.0004202 | 0.0084574 |
| CYHR1 | 0.0004202 | 0.0084574 |
| DOHH | 0.0004202 | 0.0084574 |
| DTYMK | 0.0004202 | 0.0084574 |
| ERCC6L | 0.0004202 | 0.0084574 |
| GPC1 | 0.0004202 | 0.0084574 |
| HOXB7 | 0.0004202 | 0.0084574 |
| LRTOMT | 0.0004202 | 0.0084574 |
| MNAT1 | 0.0004202 | 0.0084574 |
| MRPL35 | 0.0004202 | 0.0084574 |
| MRPS34 | 0.0004202 | 0.0084574 |
| NAE1 | 0.0004202 | 0.0084574 |
| PIAS4 | 0.0004202 | 0.0084574 |
| PTGES | 0.0004202 | 0.0084574 |
| RNF26 | 0.0004202 | 0.0084574 |
| RPL39L | 0.0004202 | 0.0084574 |
| RUVBL1 | 0.0004202 | 0.0084574 |
| SUV39H2 | 0.0004202 | 0.0084574 |
| SYMPK | 0.0004202 | 0.0084574 |
| TMEM209 | 0.0004202 | 0.0084574 |
| TSFM | 0.0004202 | 0.0084574 |
| UMPS | 0.0004202 | 0.0084574 |
| WDR76 | 0.0004202 | 0.0084574 |
| ADSL | 0.0005128 | 0.0095036 |
| AURKAIP1 | 0.0005128 | 0.0095036 |
| C1orf43 | 0.0005128 | 0.0095036 |
| DTL | 0.0005128 | 0.0095036 |
| EIF5A2 | 0.0005128 | 0.0095036 |
| LPHN1 | 0.0005128 | 0.0095036 |
| LSM14B | 0.0005128 | 0.0095036 |
| MCCC2 | 0.0005128 | 0.0095036 |
| NR1H3 | 0.0005128 | 0.0095036 |
| NUP205 | 0.0005128 | 0.0095036 |
| POLR3K | 0.0005128 | 0.0095036 |
| PSMD4 | 0.0005128 | 0.0095036 |
| SP140L | 0.0005128 | 0.0095036 |
| TCFL5 | 0.0005128 | 0.0095036 |
| TEX261 | 0.0005128 | 0.0095036 |
| TNFRSF9 | 0.0005128 | 0.0095036 |
| TTC27 | 0.0005128 | 0.0095036 |
| TTC5 | 0.0005128 | 0.0095036 |
| USP5 | 0.0005128 | 0.0095036 |
| XRCC6 | 0.0005128 | 0.0095036 |
| APEX1 | 0.0006231 | 0.0103408 |
| CACYBP | 0.0006231 | 0.0103408 |
| DHX37 | 0.0006231 | 0.0103408 |
| DNMT3B | 0.0006231 | 0.0103408 |
| DPY30 | 0.0006231 | 0.0103408 |
| GTF2H2 | 0.0006231 | 0.0103408 |
| IGHMBP2 | 0.0006231 | 0.0103408 |
| KIF20A | 0.0006231 | 0.0103408 |
| MAP6D1 | 0.0006231 | 0.0103408 |
| NDUFS6 | 0.0006231 | 0.0103408 |
| OIP5 | 0.0006231 | 0.0103408 |
| PELP1 | 0.0006231 | 0.0103408 |
| PSMA3 | 0.0006231 | 0.0103408 |
| PTCD1 | 0.0006231 | 0.0103408 |
| RFT1 | 0.0006231 | 0.0103408 |
| RRP9 | 0.0006231 | 0.0103408 |
| SAFB | 0.0006231 | 0.0103408 |
| SDCCAG3 | 0.0006231 | 0.0103408 |
| SIX4 | 0.0006231 | 0.0103408 |
| SKIL | 0.0006231 | 0.0103408 |
| SNAPC4 | 0.0006231 | 0.0103408 |
| SNRPD1 | 0.0006231 | 0.0103408 |
| TAF1B | 0.0006231 | 0.0103408 |
| TLK1 | 0.0006231 | 0.0103408 |
| TOMM20 | 0.0006231 | 0.0103408 |
| TOMM40 | 0.0006231 | 0.0103408 |
| C3orf52 | 0.0007528 | 0.011378 |
| DCAF15 | 0.0007528 | 0.011378 |
| DEPDC1 | 0.0007528 | 0.011378 |
| FBXL6 | 0.0007528 | 0.011378 |
| FGF12 | 0.0007528 | 0.011378 |
| FUS | 0.0007528 | 0.011378 |
| KIAA1549 | 0.0007528 | 0.011378 |
| L2HGDH | 0.0007528 | 0.011378 |
| MKI67 | 0.0007528 | 0.011378 |
| MTHFD1L | 0.0007528 | 0.011378 |
| NFKBIB | 0.0007528 | 0.011378 |
| PDXP | 0.0007528 | 0.011378 |
| PPHLN1 | 0.0007528 | 0.011378 |
| PRR5 | 0.0007528 | 0.011378 |
| RUNDC1 | 0.0007528 | 0.011378 |
| STRA13 | 0.0007528 | 0.011378 |
| THOC5 | 0.0007528 | 0.011378 |
| THOP1 | 0.0007528 | 0.011378 |
| TMEM206 | 0.0007528 | 0.011378 |
| TTC26 | 0.0007528 | 0.011378 |
| WDR18 | 0.0007528 | 0.011378 |
| AAGAB | 0.0009058 | 0.0124153 |
| ARHGAP8 | 0.0009058 | 0.0124153 |
| AVL9 | 0.0009058 | 0.0124153 |
| BCAS4 | 0.0009058 | 0.0124153 |
| CENPK | 0.0009058 | 0.0124153 |
| CHORDC1 | 0.0009058 | 0.0124153 |
| DAK | 0.0009058 | 0.0124153 |
| DCLRE1B | 0.0009058 | 0.0124153 |
| DDHD1 | 0.0009058 | 0.0124153 |
| FBXO22 | 0.0009058 | 0.0124153 |
| IFT52 | 0.0009058 | 0.0124153 |
| KLF16 | 0.0009058 | 0.0124153 |
| 9-Mar | 0.0009058 | 0.0124153 |
| MARS2 | 0.0009058 | 0.0124153 |
| MRPL51 | 0.0009058 | 0.0124153 |
| MSX1 | 0.0009058 | 0.0124153 |
| MSX2 | 0.0009058 | 0.0124153 |
| MTERF | 0.0009058 | 0.0124153 |
| NCAPD2 | 0.0009058 | 0.0124153 |
| NUDT16L1 | 0.0009058 | 0.0124153 |
| PHF20L1 | 0.0009058 | 0.0124153 |
| POLE2 | 0.0009058 | 0.0124153 |
| RNF121 | 0.0009058 | 0.0124153 |
| SNF8 | 0.0009058 | 0.0124153 |
| TAF1A | 0.0009058 | 0.0124153 |
| ZNF696 | 0.0009058 | 0.0124153 |
| ACBD6 | 0.0010844 | 0.0135762 |
| B3GNTL1 | 0.0010844 | 0.0135762 |
| BEND3 | 0.0010844 | 0.0135762 |
| BICD1 | 0.0010844 | 0.0135762 |
| CLN6 | 0.0010844 | 0.0135762 |
| EIF4H | 0.0010844 | 0.0135762 |
| FXN | 0.0010844 | 0.0135762 |
| GAL | 0.0010844 | 0.0135762 |
| HEATR2 | 0.0010844 | 0.0135762 |
| IL17RD | 0.0010844 | 0.0135762 |
| KHK | 0.0010844 | 0.0135762 |
| MAP3K2 | 0.0010844 | 0.0135762 |
| MRPS12 | 0.0010844 | 0.0135762 |
| MRPS5 | 0.0010844 | 0.0135762 |
| NENF | 0.0010844 | 0.0135762 |
| PHC3 | 0.0010844 | 0.0135762 |
| POC1A | 0.0010844 | 0.0135762 |
| PSMA2 | 0.0010844 | 0.0135762 |
| SENP5 | 0.0010844 | 0.0135762 |
| SRSF3 | 0.0010844 | 0.0135762 |
| SURF2 | 0.0010844 | 0.0135762 |
| TOE1 | 0.0010844 | 0.0135762 |
| TPI1 | 0.0010844 | 0.0135762 |
| TWISTNB | 0.0010844 | 0.0135762 |
| UGGT1 | 0.0010844 | 0.0135762 |
| UNKL | 0.0010844 | 0.0135762 |
| UQCRH | 0.0010844 | 0.0135762 |
| USP39 | 0.0010844 | 0.0135762 |
| ZNF670 | 0.0010844 | 0.0135762 |
| AARSD1 | 0.0012933 | 0.0147299 |
| ACOT8 | 0.0012933 | 0.0147299 |
| ACVR2B | 0.0012933 | 0.0147299 |
| AHCTF1 | 0.0012933 | 0.0147299 |
| ARHGAP11A | 0.0012933 | 0.0147299 |
| ASXL1 | 0.0012933 | 0.0147299 |
| BCL2L11 | 0.0012933 | 0.0147299 |
| C1orf27 | 0.0012933 | 0.0147299 |
| C5orf28 | 0.0012933 | 0.0147299 |
| CBS | 0.0012933 | 0.0147299 |
| CCT7 | 0.0012933 | 0.0147299 |
| CENPL | 0.0012933 | 0.0147299 |
| CKAP2 | 0.0012933 | 0.0147299 |
| DTNBP1 | 0.0012933 | 0.0147299 |
| EARS2 | 0.0012933 | 0.0147299 |
| ELK4 | 0.0012933 | 0.0147299 |
| EN1 | 0.0012933 | 0.0147299 |
| GTF2H2B | 0.0012933 | 0.0147299 |
| H2AFZ | 0.0012933 | 0.0147299 |
| HSP90AB1 | 0.0012933 | 0.0147299 |
| IPO9 | 0.0012933 | 0.0147299 |
| MRPL14 | 0.0012933 | 0.0147299 |
| MRPS15 | 0.0012933 | 0.0147299 |
| NCDN | 0.0012933 | 0.0147299 |
| NPL | 0.0012933 | 0.0147299 |
| NR2C1 | 0.0012933 | 0.0147299 |
| PIF1 | 0.0012933 | 0.0147299 |
| PLEKHG4 | 0.0012933 | 0.0147299 |
| PPP2R3C | 0.0012933 | 0.0147299 |
| PSMB1 | 0.0012933 | 0.0147299 |
| RABEPK | 0.0012933 | 0.0147299 |
| RANGAP1 | 0.0012933 | 0.0147299 |
| RRP12 | 0.0012933 | 0.0147299 |
| SLC1A5 | 0.0012933 | 0.0147299 |
| WHSC1L1 | 0.0012933 | 0.0147299 |
| AP3M2 | 0.0015354 | 0.0161252 |
| ATP5G3 | 0.0015354 | 0.0161252 |
| BYSL | 0.0015354 | 0.0161252 |
| C7orf25 | 0.0015354 | 0.0161252 |
| CBX3 | 0.0015354 | 0.0161252 |
| CDR2L | 0.0015354 | 0.0161252 |
| CHML | 0.0015354 | 0.0161252 |
| COX4I1 | 0.0015354 | 0.0161252 |
| DPY19L1 | 0.0015354 | 0.0161252 |
| FBL | 0.0015354 | 0.0161252 |
| FNDC3B | 0.0015354 | 0.0161252 |
| GNL3L | 0.0015354 | 0.0161252 |
| GSDMD | 0.0015354 | 0.0161252 |
| GSG2 | 0.0015354 | 0.0161252 |
| MARK1 | 0.0015354 | 0.0161252 |
| MRPS7 | 0.0015354 | 0.0161252 |
| NEURL4 | 0.0015354 | 0.0161252 |
| PEX10 | 0.0015354 | 0.0161252 |
| PFDN2 | 0.0015354 | 0.0161252 |
| POFUT1 | 0.0015354 | 0.0161252 |
| POLR2K | 0.0015354 | 0.0161252 |
| PSMC3IP | 0.0015354 | 0.0161252 |
| PTPDC1 | 0.0015354 | 0.0161252 |
| SBNO2 | 0.0015354 | 0.0161252 |
| STK4 | 0.0015354 | 0.0161252 |
| VEGFB | 0.0015354 | 0.0161252 |
| XCL1 | 0.0015354 | 0.0161252 |
| XCL2 | 0.0015354 | 0.0161252 |
| ZDHHC23 | 0.0015354 | 0.0161252 |
| ZNF200 | 0.0015354 | 0.0161252 |
| ZNF669 | 0.0015354 | 0.0161252 |
| CABYR | 0.0018165 | 0.0173568 |
| CCNA2 | 0.0018165 | 0.0173568 |
| CD320 | 0.0018165 | 0.0173568 |
| CENPJ | 0.0018165 | 0.0173568 |
| CKS2 | 0.0018165 | 0.0173568 |
| CXorf57 | 0.0018165 | 0.0173568 |
| DHX57 | 0.0018165 | 0.0173568 |
| DPP7 | 0.0018165 | 0.0173568 |
| GNG4 | 0.0018165 | 0.0173568 |
| GPR98 | 0.0018165 | 0.0173568 |
| H2AFY2 | 0.0018165 | 0.0173568 |
| HSPA9 | 0.0018165 | 0.0173568 |
| HSPB11 | 0.0018165 | 0.0173568 |
| IFT122 | 0.0018165 | 0.0173568 |
| LLPH | 0.0018165 | 0.0173568 |
| LRRC23 | 0.0018165 | 0.0173568 |
| MLF1 | 0.0018165 | 0.0173568 |
| MPPED2 | 0.0018165 | 0.0173568 |
| MRE11A | 0.0018165 | 0.0173568 |
| MRPL34 | 0.0018165 | 0.0173568 |
| MSTO2P | 0.0018165 | 0.0173568 |
| NECAB3 | 0.0018165 | 0.0173568 |
| NPRL3 | 0.0018165 | 0.0173568 |
| NSUN4 | 0.0018165 | 0.0173568 |
| NUP153 | 0.0018165 | 0.0173568 |
| PHTF2 | 0.0018165 | 0.0173568 |
| PRKAR1B | 0.0018165 | 0.0173568 |
| RBM41 | 0.0018165 | 0.0173568 |
| RNASET2 | 0.0018165 | 0.0173568 |
| SPPL2B | 0.0018165 | 0.0173568 |
| URB2 | 0.0018165 | 0.0173568 |
| YDJC | 0.0018165 | 0.0173568 |
| ZNF180 | 0.0018165 | 0.0173568 |
| AMPD2 | 0.0021401 | 0.0189256 |
| BAHD1 | 0.0021401 | 0.0189256 |
| CASC5 | 0.0021401 | 0.0189256 |
| CCT2 | 0.0021401 | 0.0189256 |
| CDK6 | 0.0021401 | 0.0189256 |
| COPS7B | 0.0021401 | 0.0189256 |
| COQ6 | 0.0021401 | 0.0189256 |
| EPHB2 | 0.0021401 | 0.0189256 |
| ERF | 0.0021401 | 0.0189256 |
| FAM3C | 0.0021401 | 0.0189256 |
| FAM98A | 0.0021401 | 0.0189256 |
| GALNT6 | 0.0021401 | 0.0189256 |
| GTF3C4 | 0.0021401 | 0.0189256 |
| LAGE3 | 0.0021401 | 0.0189256 |
| MED10 | 0.0021401 | 0.0189256 |
| MPHOSPH9 | 0.0021401 | 0.0189256 |
| NUBP2 | 0.0021401 | 0.0189256 |
| PABPN1 | 0.0021401 | 0.0189256 |
| PRR5-ARHGAP8 | 0.0021401 | 0.0189256 |
| RMND1 | 0.0021401 | 0.0189256 |
| RPAIN | 0.0021401 | 0.0189256 |
| SCNM1 | 0.0021401 | 0.0189256 |
| SEC22A | 0.0021401 | 0.0189256 |
| ST3GAL2 | 0.0021401 | 0.0189256 |
| TFG | 0.0021401 | 0.0189256 |
| TRIM11 | 0.0021401 | 0.0189256 |
| TRMT61A | 0.0021401 | 0.0189256 |
| UBE2I | 0.0021401 | 0.0189256 |
| YEATS2 | 0.0021401 | 0.0189256 |
| YTHDF1 | 0.0021401 | 0.0189256 |
| YWHAQ | 0.0021401 | 0.0189256 |
| ZNF747 | 0.0021401 | 0.0189256 |
| ACP1 | 0.0025129 | 0.0207024 |
| ACTR3B | 0.0025129 | 0.0207024 |
| ARL17A | 0.0025129 | 0.0207024 |
| ARMC7 | 0.0025129 | 0.0207024 |
| ATP5G1 | 0.0025129 | 0.0207024 |
| ATPAF2 | 0.0025129 | 0.0207024 |
| C19orf24 | 0.0025129 | 0.0207024 |
| C20orf24 | 0.0025129 | 0.0207024 |
| C5orf22 | 0.0025129 | 0.0207024 |
| CENPI | 0.0025129 | 0.0207024 |
| CTNS | 0.0025129 | 0.0207024 |
| DCAF7 | 0.0025129 | 0.0207024 |
| DGUOK | 0.0025129 | 0.0207024 |
| FAM133B | 0.0025129 | 0.0207024 |
| FZR1 | 0.0025129 | 0.0207024 |
| GFER | 0.0025129 | 0.0207024 |
| GNAS | 0.0025129 | 0.0207024 |
| GTPBP10 | 0.0025129 | 0.0207024 |
| KREMEN2 | 0.0025129 | 0.0207024 |
| MOGS | 0.0025129 | 0.0207024 |
| MRPS30 | 0.0025129 | 0.0207024 |
| MTOR | 0.0025129 | 0.0207024 |
| PDCD2L | 0.0025129 | 0.0207024 |
| PDSS1 | 0.0025129 | 0.0207024 |
| PIGM | 0.0025129 | 0.0207024 |
| RTN4IP1 | 0.0025129 | 0.0207024 |
| SLC16A10 | 0.0025129 | 0.0207024 |
| SNCAIP | 0.0025129 | 0.0207024 |
| SNRPD2 | 0.0025129 | 0.0207024 |
| SOX18 | 0.0025129 | 0.0207024 |
| SUZ12 | 0.0025129 | 0.0207024 |
| TMEM70 | 0.0025129 | 0.0207024 |
| TTLL4 | 0.0025129 | 0.0207024 |
| USP42 | 0.0025129 | 0.0207024 |
| ABTB2 | 0.0029396 | 0.0221725 |
| ANKH | 0.0029396 | 0.0221725 |
| B3GAT3 | 0.0029396 | 0.0221725 |
| BNIP1 | 0.0029396 | 0.0221725 |
| BUD31 | 0.0029396 | 0.0221725 |
| CASC4 | 0.0029396 | 0.0221725 |
| CTTN | 0.0029396 | 0.0221725 |
| DDX20 | 0.0029396 | 0.0221725 |
| DYNC1I1 | 0.0029396 | 0.0221725 |
| EEF1E1 | 0.0029396 | 0.0221725 |
| EGF | 0.0029396 | 0.0221725 |
| EIF3H | 0.0029396 | 0.0221725 |
| FER1L4 | 0.0029396 | 0.0221725 |
| GRAPL | 0.0029396 | 0.0221725 |
| HNRNPH3 | 0.0029396 | 0.0221725 |
| HRSP12 | 0.0029396 | 0.0221725 |
| MBOAT7 | 0.0029396 | 0.0221725 |
| MRPL13 | 0.0029396 | 0.0221725 |
| MSTO1 | 0.0029396 | 0.0221725 |
| NAA10 | 0.0029396 | 0.0221725 |
| NHLRC2 | 0.0029396 | 0.0221725 |
| NSD1 | 0.0029396 | 0.0221725 |
| OGFOD2 | 0.0029396 | 0.0221725 |
| PHF14 | 0.0029396 | 0.0221725 |
| POMT2 | 0.0029396 | 0.0221725 |
| PRDX1 | 0.0029396 | 0.0221725 |
| RPN2 | 0.0029396 | 0.0221725 |
| SIRT5 | 0.0029396 | 0.0221725 |
| SMYD4 | 0.0029396 | 0.0221725 |
| SNRPF | 0.0029396 | 0.0221725 |
| TMEM199 | 0.0029396 | 0.0221725 |
| TMEM39A | 0.0029396 | 0.0221725 |
| UTP15 | 0.0029396 | 0.0221725 |
| WDR46 | 0.0029396 | 0.0221725 |
| YIPF4 | 0.0029396 | 0.0221725 |
| ZNF813 | 0.0029396 | 0.0221725 |
| ZSCAN5A | 0.0029396 | 0.0221725 |
| AKR7A3 | 0.0034279 | 0.0238422 |
| ARMC5 | 0.0034279 | 0.0238422 |
| ASB6 | 0.0034279 | 0.0238422 |
| B3GALT6 | 0.0034279 | 0.0238422 |
| CARS2 | 0.0034279 | 0.0238422 |
| CIDEC | 0.0034279 | 0.0238422 |
| CNBP | 0.0034279 | 0.0238422 |
| CPT1A | 0.0034279 | 0.0238422 |
| CUL4A | 0.0034279 | 0.0238422 |
| CWF19L1 | 0.0034279 | 0.0238422 |
| DCTPP1 | 0.0034279 | 0.0238422 |
| DGCR14 | 0.0034279 | 0.0238422 |
| FBXW2 | 0.0034279 | 0.0238422 |
| GLRX5 | 0.0034279 | 0.0238422 |
| GTF2A2 | 0.0034279 | 0.0238422 |
| HMGB3 | 0.0034279 | 0.0238422 |
| ITGB1BP1 | 0.0034279 | 0.0238422 |
| MTHFSD | 0.0034279 | 0.0238422 |
| NHP2L1 | 0.0034279 | 0.0238422 |
| NME1-NME2 | 0.0034279 | 0.0238422 |
| NME2 | 0.0034279 | 0.0238422 |
| NUP62 | 0.0034279 | 0.0238422 |
| NUP62CL | 0.0034279 | 0.0238422 |
| PHF6 | 0.0034279 | 0.0238422 |
| PRR7 | 0.0034279 | 0.0238422 |
| RANGRF | 0.0034279 | 0.0238422 |
| RBKS | 0.0034279 | 0.0238422 |
| RBL1 | 0.0034279 | 0.0238422 |
| SLC12A8 | 0.0034279 | 0.0238422 |
| SLC35B2 | 0.0034279 | 0.0238422 |
| SMG5 | 0.0034279 | 0.0238422 |
| STAU2 | 0.0034279 | 0.0238422 |
| TEX10 | 0.0034279 | 0.0238422 |
| TOR3A | 0.0034279 | 0.0238422 |
| UBE2W | 0.0034279 | 0.0238422 |
| XRCC2 | 0.0034279 | 0.0238422 |
| YRDC | 0.0034279 | 0.0238422 |
| ZNF607 | 0.0034279 | 0.0238422 |
| ANGEL2 | 0.0039833 | 0.0259142 |
| ANKRD16 | 0.0039833 | 0.0259142 |
| AP1S3 | 0.0039833 | 0.0259142 |
| ARPC1A | 0.0039833 | 0.0259142 |
| B4GALT7 | 0.0039833 | 0.0259142 |
| C11orf24 | 0.0039833 | 0.0259142 |
| C4orf29 | 0.0039833 | 0.0259142 |
| C9orf114 | 0.0039833 | 0.0259142 |
| CD72 | 0.0039833 | 0.0259142 |
| ENDOV | 0.0039833 | 0.0259142 |
| ENTPD7 | 0.0039833 | 0.0259142 |
| FAM195A | 0.0039833 | 0.0259142 |
| GABRR1 | 0.0039833 | 0.0259142 |
| GDF15 | 0.0039833 | 0.0259142 |
| HM13 | 0.0039833 | 0.0259142 |
| HNRNPA2B1 | 0.0039833 | 0.0259142 |
| INPP4A | 0.0039833 | 0.0259142 |
| LDHA | 0.0039833 | 0.0259142 |
| LPHN3 | 0.0039833 | 0.0259142 |
| MKS1 | 0.0039833 | 0.0259142 |
| MMP11 | 0.0039833 | 0.0259142 |
| PCBP2 | 0.0039833 | 0.0259142 |
| PEX11A | 0.0039833 | 0.0259142 |
| RAPGEF4 | 0.0039833 | 0.0259142 |
| SIKE1 | 0.0039833 | 0.0259142 |
| SQSTM1 | 0.0039833 | 0.0259142 |
| TRMT2B | 0.0039833 | 0.0259142 |
| TUFM | 0.0039833 | 0.0259142 |
| ZMYM3 | 0.0039833 | 0.0259142 |
| ZNF286A | 0.0039833 | 0.0259142 |
| ZNF618 | 0.0039833 | 0.0259142 |
| AEBP2 | 0.0046148 | 0.0282868 |
| ALG10 | 0.0046148 | 0.0282868 |
| ALMS1 | 0.0046148 | 0.0282868 |
| ANKRD32 | 0.0046148 | 0.0282868 |
| ATRIP | 0.0046148 | 0.0282868 |
| BEST3 | 0.0046148 | 0.0282868 |
| BOLA1 | 0.0046148 | 0.0282868 |
| C11orf48 | 0.0046148 | 0.0282868 |
| C2orf44 | 0.0046148 | 0.0282868 |
| CCDC91 | 0.0046148 | 0.0282868 |
| CCDC93 | 0.0046148 | 0.0282868 |
| CDCA7 | 0.0046148 | 0.0282868 |
| CLNS1A | 0.0046148 | 0.0282868 |
| CSNK1D | 0.0046148 | 0.0282868 |
| FANCF | 0.0046148 | 0.0282868 |
| FHOD1 | 0.0046148 | 0.0282868 |
| INPP5K | 0.0046148 | 0.0282868 |
| KLHL12 | 0.0046148 | 0.0282868 |
| LMBR1 | 0.0046148 | 0.0282868 |
| METAP2 | 0.0046148 | 0.0282868 |
| MTA3 | 0.0046148 | 0.0282868 |
| NOD1 | 0.0046148 | 0.0282868 |
| PIP4K2B | 0.0046148 | 0.0282868 |
| PRR12 | 0.0046148 | 0.0282868 |
| PSMA1 | 0.0046148 | 0.0282868 |
| PSMA5 | 0.0046148 | 0.0282868 |
| PTGR2 | 0.0046148 | 0.0282868 |
| RAB26 | 0.0046148 | 0.0282868 |
| RBBP9 | 0.0046148 | 0.0282868 |
| SPSB2 | 0.0046148 | 0.0282868 |
| SUPT3H | 0.0046148 | 0.0282868 |
| TUBGCP2 | 0.0046148 | 0.0282868 |
| ZNF174 | 0.0046148 | 0.0282868 |
| AKR1E2 | 0.0053292 | 0.0304583 |
| C19orf43 | 0.0053292 | 0.0304583 |
| CANX | 0.0053292 | 0.0304583 |
| CBFA2T2 | 0.0053292 | 0.0304583 |
| CDK10 | 0.0053292 | 0.0304583 |
| CHCHD2 | 0.0053292 | 0.0304583 |
| CNPY2 | 0.0053292 | 0.0304583 |
| EIF2S2 | 0.0053292 | 0.0304583 |
| EN2 | 0.0053292 | 0.0304583 |
| ERMAP | 0.0053292 | 0.0304583 |
| FAM120A | 0.0053292 | 0.0304583 |
| FAM168B | 0.0053292 | 0.0304583 |
| HEATR6 | 0.0053292 | 0.0304583 |
| HFE | 0.0053292 | 0.0304583 |
| HMMR | 0.0053292 | 0.0304583 |
| HSF1 | 0.0053292 | 0.0304583 |
| LARP1B | 0.0053292 | 0.0304583 |
| NAT9 | 0.0053292 | 0.0304583 |
| PEG10 | 0.0053292 | 0.0304583 |
| PSMD7 | 0.0053292 | 0.0304583 |
| PSMG3 | 0.0053292 | 0.0304583 |
| REL | 0.0053292 | 0.0304583 |
| RNASEH2C | 0.0053292 | 0.0304583 |
| RPUSD1 | 0.0053292 | 0.0304583 |
| SHMT2 | 0.0053292 | 0.0304583 |
| SMYD2 | 0.0053292 | 0.0304583 |
| SORD | 0.0053292 | 0.0304583 |
| SPHK2 | 0.0053292 | 0.0304583 |
| SUMO1 | 0.0053292 | 0.0304583 |
| TACR1 | 0.0053292 | 0.0304583 |
| TBP | 0.0053292 | 0.0304583 |
| TCEB1 | 0.0053292 | 0.0304583 |
| TECR | 0.0053292 | 0.0304583 |
| TGFBRAP1 | 0.0053292 | 0.0304583 |
| TIMM50 | 0.0053292 | 0.0304583 |
| TRAF4 | 0.0053292 | 0.0304583 |
| ALG5 | 0.0061368 | 0.0330538 |
| AP1AR | 0.0061368 | 0.0330538 |
| C11orf30 | 0.0061368 | 0.0330538 |
| C21orf119 | 0.0061368 | 0.0330538 |
| CDC123 | 0.0061368 | 0.0330538 |
| CDKL1 | 0.0061368 | 0.0330538 |
| FA2H | 0.0061368 | 0.0330538 |
| HN1 | 0.0061368 | 0.0330538 |
| HOXA9 | 0.0061368 | 0.0330538 |
| KIAA0391 | 0.0061368 | 0.0330538 |
| KIAA0513 | 0.0061368 | 0.0330538 |
| LEF1 | 0.0061368 | 0.0330538 |
| LRRC8E | 0.0061368 | 0.0330538 |
| NAA38 | 0.0061368 | 0.0330538 |
| ORMDL1 | 0.0061368 | 0.0330538 |
| PRAME | 0.0061368 | 0.0330538 |
| PRICKLE1 | 0.0061368 | 0.0330538 |
| RAVER1 | 0.0061368 | 0.0330538 |
| RNASEH2A | 0.0061368 | 0.0330538 |
| SLC15A2 | 0.0061368 | 0.0330538 |
| SLC25A5 | 0.0061368 | 0.0330538 |
| TIMM22 | 0.0061368 | 0.0330538 |
| TMEM68 | 0.0061368 | 0.0330538 |
| TRIM37 | 0.0061368 | 0.0330538 |
| UBE2J2 | 0.0061368 | 0.0330538 |
| USP20 | 0.0061368 | 0.0330538 |
| VAV2 | 0.0061368 | 0.0330538 |
| WDR53 | 0.0061368 | 0.0330538 |
| WDR83 | 0.0061368 | 0.0330538 |
| ZNHIT2 | 0.0061368 | 0.0330538 |
| ABCC4 | 0.0070452 | 0.0361116 |
| ATAD5 | 0.0070452 | 0.0361116 |
| CCDC101 | 0.0070452 | 0.0361116 |
| CCDC86 | 0.0070452 | 0.0361116 |
| DMRT2 | 0.0070452 | 0.0361116 |
| DOCK6 | 0.0070452 | 0.0361116 |
| GATA2 | 0.0070452 | 0.0361116 |
| GPR137C | 0.0070452 | 0.0361116 |
| GTF2IRD2 | 0.0070452 | 0.0361116 |
| MAP7D3 | 0.0070452 | 0.0361116 |
| NEB | 0.0070452 | 0.0361116 |
| NFYA | 0.0070452 | 0.0361116 |
| NRIP3 | 0.0070452 | 0.0361116 |
| NUP43 | 0.0070452 | 0.0361116 |
| RNF2 | 0.0070452 | 0.0361116 |
| RNFT1 | 0.0070452 | 0.0361116 |
| RPAP1 | 0.0070452 | 0.0361116 |
| SLC23A2 | 0.0070452 | 0.0361116 |
| TADA2A | 0.0070452 | 0.0361116 |
| TSPAN33 | 0.0070452 | 0.0361116 |
| TTN | 0.0070452 | 0.0361116 |
| TUBGCP4 | 0.0070452 | 0.0361116 |
| ZNF107 | 0.0070452 | 0.0361116 |
| ZNF26 | 0.0070452 | 0.0361116 |
| ACPT | 0.0080663 | 0.0389832 |
| ANKRD26 | 0.0080663 | 0.0389832 |
| ATAD2B | 0.0080663 | 0.0389832 |
| ATL2 | 0.0080663 | 0.0389832 |
| ATP5B | 0.0080663 | 0.0389832 |
| BCKDK | 0.0080663 | 0.0389832 |
| CCDC88A | 0.0080663 | 0.0389832 |
| CINP | 0.0080663 | 0.0389832 |
| COMMD4 | 0.0080663 | 0.0389832 |
| DCAKD | 0.0080663 | 0.0389832 |
| EPRS | 0.0080663 | 0.0389832 |
| FGFR1OP | 0.0080663 | 0.0389832 |
| FPGS | 0.0080663 | 0.0389832 |
| GJC1 | 0.0080663 | 0.0389832 |
| GPATCH2 | 0.0080663 | 0.0389832 |
| HERC5 | 0.0080663 | 0.0389832 |
| HTATSF1 | 0.0080663 | 0.0389832 |
| ICA1L | 0.0080663 | 0.0389832 |
| KIAA1429 | 0.0080663 | 0.0389832 |
| LTV1 | 0.0080663 | 0.0389832 |
| MRPL45 | 0.0080663 | 0.0389832 |
| NARG2 | 0.0080663 | 0.0389832 |
| NOC4L | 0.0080663 | 0.0389832 |
| PANX2 | 0.0080663 | 0.0389832 |
| PRKD3 | 0.0080663 | 0.0389832 |
| RBM45 | 0.0080663 | 0.0389832 |
| SASS6 | 0.0080663 | 0.0389832 |
| SIPA1 | 0.0080663 | 0.0389832 |
| SLC16A5 | 0.0080663 | 0.0389832 |
| SNRPD3 | 0.0080663 | 0.0389832 |
| TBCD | 0.0080663 | 0.0389832 |
| TMEM38B | 0.0080663 | 0.0389832 |
| USP25 | 0.0080663 | 0.0389832 |
| ZBTB24 | 0.0080663 | 0.0389832 |
| ZNF124 | 0.0080663 | 0.0389832 |
| ZNF592 | 0.0080663 | 0.0389832 |
| ACAD10 | 0.0092091 | 0.0415037 |
| ALG10B | 0.0092091 | 0.0415037 |
| ALKBH1 | 0.0092091 | 0.0415037 |
| APRT | 0.0092091 | 0.0415037 |
| ARMCX4 | 0.0092091 | 0.0415037 |
| BAG2 | 0.0092091 | 0.0415037 |
| BCL7A | 0.0092091 | 0.0415037 |
| CASP8AP2 | 0.0092091 | 0.0415037 |
| CXCL10 | 0.0092091 | 0.0415037 |
| CXCL3 | 0.0092091 | 0.0415037 |
| DCUN1D2 | 0.0092091 | 0.0415037 |
| DUS3L | 0.0092091 | 0.0415037 |
| ERGIC2 | 0.0092091 | 0.0415037 |
| EXOSC7 | 0.0092091 | 0.0415037 |
| FLAD1 | 0.0092091 | 0.0415037 |
| GART | 0.0092091 | 0.0415037 |
| GDAP2 | 0.0092091 | 0.0415037 |
| GLRX3 | 0.0092091 | 0.0415037 |
| GRB14 | 0.0092091 | 0.0415037 |
| JRK | 0.0092091 | 0.0415037 |
| KCTD2 | 0.0092091 | 0.0415037 |
| KIAA1462 | 0.0092091 | 0.0415037 |
| KIAA1586 | 0.0092091 | 0.0415037 |
| LSM10 | 0.0092091 | 0.0415037 |
| METTL4 | 0.0092091 | 0.0415037 |
| NGEF | 0.0092091 | 0.0415037 |
| NIN | 0.0092091 | 0.0415037 |
| NSUN6 | 0.0092091 | 0.0415037 |
| PCBD2 | 0.0092091 | 0.0415037 |
| PCSK6 | 0.0092091 | 0.0415037 |
| PON3 | 0.0092091 | 0.0415037 |
| PRKRA | 0.0092091 | 0.0415037 |
| RANBP3 | 0.0092091 | 0.0415037 |
| RIC8B | 0.0092091 | 0.0415037 |
| RPL8 | 0.0092091 | 0.0415037 |
| SDF2L1 | 0.0092091 | 0.0415037 |
| SENP1 | 0.0092091 | 0.0415037 |
| SLC25A22 | 0.0092091 | 0.0415037 |
| SNIP1 | 0.0092091 | 0.0415037 |
| SPCS2 | 0.0092091 | 0.0415037 |
| TBC1D15 | 0.0092091 | 0.0415037 |
| TIMM44 | 0.0092091 | 0.0415037 |
| TMEM107 | 0.0092091 | 0.0415037 |
| TMUB1 | 0.0092091 | 0.0415037 |
| TNFRSF10D | 0.0092091 | 0.0415037 |
| TRIM36 | 0.0092091 | 0.0415037 |
| TRPM2 | 0.0092091 | 0.0415037 |
| TUBA1B | 0.0092091 | 0.0415037 |
| TYRO3 | 0.0092091 | 0.0415037 |
| WDR54 | 0.0092091 | 0.0415037 |
| XAB2 | 0.0092091 | 0.0415037 |
| C1orf61 | 0.0104869 | 0.0448231 |
| CALB1 | 0.0104869 | 0.0448231 |
| CCNJ | 0.0104869 | 0.0448231 |
| CDC25B | 0.0104869 | 0.0448231 |
| CERCAM | 0.0104869 | 0.0448231 |
| CYP26B1 | 0.0104869 | 0.0448231 |
| DAG1 | 0.0104869 | 0.0448231 |
| FAM162A | 0.0104869 | 0.0448231 |
| FUT1 | 0.0104869 | 0.0448231 |
| IQCG | 0.0104869 | 0.0448231 |
| KIAA1244 | 0.0104869 | 0.0448231 |
| KRR1 | 0.0104869 | 0.0448231 |
| LOXL3 | 0.0104869 | 0.0448231 |
| LRPAP1 | 0.0104869 | 0.0448231 |
| MED26 | 0.0104869 | 0.0448231 |
| NAPEPLD | 0.0104869 | 0.0448231 |
| NCLN | 0.0104869 | 0.0448231 |
| NOS1AP | 0.0104869 | 0.0448231 |
| PDCD6 | 0.0104869 | 0.0448231 |
| PLK4 | 0.0104869 | 0.0448231 |
| POMT1 | 0.0104869 | 0.0448231 |
| PPARG | 0.0104869 | 0.0448231 |
| PQBP1 | 0.0104869 | 0.0448231 |
| PSMD3 | 0.0104869 | 0.0448231 |
| RAB34 | 0.0104869 | 0.0448231 |
| SH3BP2 | 0.0104869 | 0.0448231 |
| SLC5A12 | 0.0104869 | 0.0448231 |
| SRSF9 | 0.0104869 | 0.0448231 |
| SYT12 | 0.0104869 | 0.0448231 |
| TAF9 | 0.0104869 | 0.0448231 |
| THEM4 | 0.0104869 | 0.0448231 |
| TMX2 | 0.0104869 | 0.0448231 |
| TRPM4 | 0.0104869 | 0.0448231 |
| TSKS | 0.0104869 | 0.0448231 |
| TSPAN9 | 0.0104869 | 0.0448231 |
| XPR1 | 0.0104869 | 0.0448231 |
| ZCCHC4 | 0.0104869 | 0.0448231 |
| ZNF239 | 0.0104869 | 0.0448231 |
| ZNF253 | 0.0104869 | 0.0448231 |
| ZNF480 | 0.0104869 | 0.0448231 |
| ZNF599 | 0.0104869 | 0.0448231 |
| ABHD6 | 0.0119098 | 0.0484313 |
| ADAM22 | 0.0119098 | 0.0484313 |
| ADCK1 | 0.0119098 | 0.0484313 |
| ARF1 | 0.0119098 | 0.0484313 |
| C15orf39 | 0.0119098 | 0.0484313 |
| C19orf25 | 0.0119098 | 0.0484313 |
| CALML4 | 0.0119098 | 0.0484313 |
| CCNE2 | 0.0119098 | 0.0484313 |
| CHMP1B | 0.0119098 | 0.0484313 |
| DDX5 | 0.0119098 | 0.0484313 |
| EFHD2 | 0.0119098 | 0.0484313 |
| FH | 0.0119098 | 0.0484313 |
| FRK | 0.0119098 | 0.0484313 |
| GZF1 | 0.0119098 | 0.0484313 |
| ICMT | 0.0119098 | 0.0484313 |
| KNTC1 | 0.0119098 | 0.0484313 |
| LARP6 | 0.0119098 | 0.0484313 |
| LIMK1 | 0.0119098 | 0.0484313 |
| LIMS1 | 0.0119098 | 0.0484313 |
| NEK6 | 0.0119098 | 0.0484313 |
| PGAP1 | 0.0119098 | 0.0484313 |
| POP5 | 0.0119098 | 0.0484313 |
| PPFIA3 | 0.0119098 | 0.0484313 |
| PRSS21 | 0.0119098 | 0.0484313 |
| RNF208 | 0.0119098 | 0.0484313 |
| SAFB2 | 0.0119098 | 0.0484313 |
| SCLY | 0.0119098 | 0.0484313 |
| SEC61G | 0.0119098 | 0.0484313 |
| SESN3 | 0.0119098 | 0.0484313 |
| SFMBT1 | 0.0119098 | 0.0484313 |
| ST20 | 0.0119098 | 0.0484313 |
| SYCP2 | 0.0119098 | 0.0484313 |
| SYT17 | 0.0119098 | 0.0484313 |
| TIMM23 | 0.0119098 | 0.0484313 |
| TUBA1C | 0.0119098 | 0.0484313 |
| USP18 | 0.0119098 | 0.0484313 |
| ZNF16 | 0.0119098 | 0.0484313 |
| ZNF165 | 0.0119098 | 0.0484313 |
| **Down-regulated genes** |  |  |
| THSD4 | 8.50E-08 | 0.0002252 |
| AHNAK | 5.95E-07 | 0.0004729 |
| SIM2 | 5.95E-07 | 0.0004729 |
| KCNAB1 | 1.02E-06 | 0.0005405 |
| IL33 | 2.55E-06 | 0.0008812 |
| TMEM40 | 2.55E-06 | 0.0008812 |
| CD59 | 5.69E-06 | 0.0015608 |
| PCP4 | 5.69E-06 | 0.0015608 |
| ABLIM3 | 8.16E-06 | 0.0016629 |
| IL11RA | 8.16E-06 | 0.0016629 |
| PPP1R3B | 8.16E-06 | 0.0016629 |
| ANXA3 | 1.16E-05 | 0.0019141 |
| MGLL | 1.16E-05 | 0.0019141 |
| SMTN | 1.16E-05 | 0.0019141 |
| ARMCX2 | 1.60E-05 | 0.002016 |
| FAM107A | 1.60E-05 | 0.002016 |
| KLK13 | 1.60E-05 | 0.002016 |
| USP9Y | 1.60E-05 | 0.002016 |
| ZNF185 | 1.60E-05 | 0.002016 |
| CAB39L | 2.19E-05 | 0.0021518 |
| EIF1AY | 2.19E-05 | 0.0021518 |
| IGF1 | 2.19E-05 | 0.0021518 |
| ITM2A | 2.19E-05 | 0.0021518 |
| LRRK2 | 2.19E-05 | 0.0021518 |
| VPS4B | 2.19E-05 | 0.0021518 |
| ARL6IP5 | 2.95E-05 | 0.0023679 |
| EPB41L3 | 2.95E-05 | 0.0023679 |
| FOXP1 | 2.95E-05 | 0.0023679 |
| MAOB | 2.95E-05 | 0.0023679 |
| MDFIC | 2.95E-05 | 0.0023679 |
| PLP1 | 2.95E-05 | 0.0023679 |
| RHCG | 2.95E-05 | 0.0023679 |
| TMEM220 | 2.95E-05 | 0.0023679 |
| DDX3Y | 3.93E-05 | 0.002585 |
| ECM1 | 3.93E-05 | 0.002585 |
| KDM5D | 3.93E-05 | 0.002585 |
| PCP4L1 | 3.93E-05 | 0.002585 |
| PMM1 | 3.93E-05 | 0.002585 |
| PPM1K | 3.93E-05 | 0.002585 |
| PTK6 | 3.93E-05 | 0.002585 |
| SRPX | 3.93E-05 | 0.002585 |
| SYNGR1 | 3.93E-05 | 0.002585 |
| CAPNS2 | 5.18E-05 | 0.0030476 |
| SDCBP2 | 5.18E-05 | 0.0030476 |
| TLR3 | 5.18E-05 | 0.0030476 |
| TMOD2 | 5.18E-05 | 0.0030476 |
| ADAMTS1 | 6.76E-05 | 0.0034875 |
| ARMCX1 | 6.76E-05 | 0.0034875 |
| DES | 6.76E-05 | 0.0034875 |
| ELL2 | 6.76E-05 | 0.0034875 |
| PPP3CC | 6.76E-05 | 0.0034875 |
| SFRP1 | 6.76E-05 | 0.0034875 |
| ZNF654 | 6.76E-05 | 0.0034875 |
| DUOXA1 | 8.71E-05 | 0.0041714 |
| SESN1 | 8.71E-05 | 0.0041714 |
| TSPAN31 | 8.71E-05 | 0.0041714 |
| C2orf40 | 0.0001116 | 0.0046932 |
| CCL21 | 0.0001116 | 0.0046932 |
| GNG11 | 0.0001116 | 0.0046932 |
| HEMK1 | 0.0001116 | 0.0046932 |
| HMGCS1 | 0.0001116 | 0.0046932 |
| MRVI1 | 0.0001116 | 0.0046932 |
| PTGS1 | 0.0001116 | 0.0046932 |
| SBSN | 0.0001116 | 0.0046932 |
| TNXB | 0.0001116 | 0.0046932 |
| ACTG2 | 0.0001415 | 0.0054078 |
| CD82 | 0.0001415 | 0.0054078 |
| PRRG1 | 0.0001415 | 0.0054078 |
| TNXA | 0.0001415 | 0.0054078 |
| ATP8A1 | 0.0001784 | 0.0058354 |
| ETHE1 | 0.0001784 | 0.0058354 |
| F13A1 | 0.0001784 | 0.0058354 |
| IL1RN | 0.0001784 | 0.0058354 |
| MAST4 | 0.0001784 | 0.0058354 |
| MEIS1 | 0.0001784 | 0.0058354 |
| PCDH18 | 0.0001784 | 0.0058354 |
| PRELP | 0.0001784 | 0.0058354 |
| RRAD | 0.0001784 | 0.0058354 |
| SLC9A9 | 0.0001784 | 0.0058354 |
| SORL1 | 0.0001784 | 0.0058354 |
| UNC13B | 0.0001784 | 0.0058354 |
| UTRN | 0.0001784 | 0.0058354 |
| YTHDC2 | 0.0001784 | 0.0058354 |
| ZBTB16 | 0.0001784 | 0.0058354 |
| ZNF665 | 0.0001784 | 0.0058354 |
| ANXA8 | 0.000223 | 0.006331 |
| ANXA8L1 | 0.000223 | 0.006331 |
| C10orf10 | 0.000223 | 0.006331 |
| CLEC3B | 0.000223 | 0.006331 |
| MTMR10 | 0.000223 | 0.006331 |
| P2RY14 | 0.000223 | 0.006331 |
| PRKAR1A | 0.000223 | 0.006331 |
| RBPMS | 0.000223 | 0.006331 |
| RPS4Y1 | 0.000223 | 0.006331 |
| RSPH3 | 0.000223 | 0.006331 |
| S1PR1 | 0.000223 | 0.006331 |
| SLC7A2 | 0.000223 | 0.006331 |
| TMEM98 | 0.000223 | 0.006331 |
| TTC28 | 0.000223 | 0.006331 |
| VAMP4 | 0.000223 | 0.006331 |
| ABAT | 0.0002772 | 0.0069959 |
| AMY2B | 0.0002772 | 0.0069959 |
| CITED2 | 0.0002772 | 0.0069959 |
| CKMT1A | 0.0002772 | 0.0069959 |
| CKMT1B | 0.0002772 | 0.0069959 |
| EDN3 | 0.0002772 | 0.0069959 |
| FILIP1L | 0.0002772 | 0.0069959 |
| HOXA3 | 0.0002772 | 0.0069959 |
| LHFP | 0.0002772 | 0.0069959 |
| LIFR | 0.0002772 | 0.0069959 |
| LXN | 0.0002772 | 0.0069959 |
| PDCD6IP | 0.0002772 | 0.0069959 |
| PDLIM5 | 0.0002772 | 0.0069959 |
| PDZRN3 | 0.0002772 | 0.0069959 |
| RNF39 | 0.0002772 | 0.0069959 |
| TP53I3 | 0.0002772 | 0.0069959 |
| ZCCHC6 | 0.0002772 | 0.0069959 |
| ANKRD6 | 0.0003421 | 0.0078156 |
| AOC3 | 0.0003421 | 0.0078156 |
| CCL19 | 0.0003421 | 0.0078156 |
| DHRS1 | 0.0003421 | 0.0078156 |
| GAS7 | 0.0003421 | 0.0078156 |
| IDS | 0.0003421 | 0.0078156 |
| KDM3A | 0.0003421 | 0.0078156 |
| LPIN1 | 0.0003421 | 0.0078156 |
| LTBP2 | 0.0003421 | 0.0078156 |
| PLEKHA5 | 0.0003421 | 0.0078156 |
| SDR16C5 | 0.0003421 | 0.0078156 |
| SH3BGRL | 0.0003421 | 0.0078156 |
| SMAD5 | 0.0003421 | 0.0078156 |
| TGFB1I1 | 0.0003421 | 0.0078156 |
| TLR5 | 0.0003421 | 0.0078156 |
| XYLT1 | 0.0003421 | 0.0078156 |
| ZFYVE21 | 0.0003421 | 0.0078156 |
| ADARB1 | 0.0004202 | 0.0084574 |
| AFF1 | 0.0004202 | 0.0084574 |
| B3GALT4 | 0.0004202 | 0.0084574 |
| CLDN11 | 0.0004202 | 0.0084574 |
| CNN1 | 0.0004202 | 0.0084574 |
| COL21A1 | 0.0004202 | 0.0084574 |
| CTTNBP2NL | 0.0004202 | 0.0084574 |
| CUL3 | 0.0004202 | 0.0084574 |
| ENDOD1 | 0.0004202 | 0.0084574 |
| EPHX2 | 0.0004202 | 0.0084574 |
| HCFC2 | 0.0004202 | 0.0084574 |
| HMGCR | 0.0004202 | 0.0084574 |
| JAM2 | 0.0004202 | 0.0084574 |
| KCTD12 | 0.0004202 | 0.0084574 |
| KLF6 | 0.0004202 | 0.0084574 |
| KLK7 | 0.0004202 | 0.0084574 |
| LTBP4 | 0.0004202 | 0.0084574 |
| MEIS3P1 | 0.0004202 | 0.0084574 |
| METTL7A | 0.0004202 | 0.0084574 |
| MTSS1 | 0.0004202 | 0.0084574 |
| OLFML3 | 0.0004202 | 0.0084574 |
| PITX1 | 0.0004202 | 0.0084574 |
| SLC43A3 | 0.0004202 | 0.0084574 |
| VAT1 | 0.0004202 | 0.0084574 |
| DIXDC1 | 0.0005128 | 0.0095036 |
| FRMD4B | 0.0005128 | 0.0095036 |
| GRN | 0.0005128 | 0.0095036 |
| GYPC | 0.0005128 | 0.0095036 |
| HPGD | 0.0005128 | 0.0095036 |
| LANCL1 | 0.0005128 | 0.0095036 |
| PPP3CB | 0.0005128 | 0.0095036 |
| PRDM1 | 0.0005128 | 0.0095036 |
| SLC6A1 | 0.0005128 | 0.0095036 |
| TCN1 | 0.0005128 | 0.0095036 |
| TIAM1 | 0.0005128 | 0.0095036 |
| TMPRSS11D | 0.0005128 | 0.0095036 |
| TPM1 | 0.0005128 | 0.0095036 |
| TSPYL2 | 0.0005128 | 0.0095036 |
| APBB1IP | 0.0006231 | 0.0103408 |
| CCDC69 | 0.0006231 | 0.0103408 |
| CCND2 | 0.0006231 | 0.0103408 |
| CNRIP1 | 0.0006231 | 0.0103408 |
| CYLD | 0.0006231 | 0.0103408 |
| DAAM2 | 0.0006231 | 0.0103408 |
| EFEMP1 | 0.0006231 | 0.0103408 |
| FAM110C | 0.0006231 | 0.0103408 |
| FAM114A1 | 0.0006231 | 0.0103408 |
| FBXL5 | 0.0006231 | 0.0103408 |
| GJB2 | 0.0006231 | 0.0103408 |
| ITGA1 | 0.0006231 | 0.0103408 |
| ITGA5 | 0.0006231 | 0.0103408 |
| JMJD7-PLA2G4B | 0.0006231 | 0.0103408 |
| KLK11 | 0.0006231 | 0.0103408 |
| MAML3 | 0.0006231 | 0.0103408 |
| MMRN1 | 0.0006231 | 0.0103408 |
| PDGFRA | 0.0006231 | 0.0103408 |
| PLA2G4B | 0.0006231 | 0.0103408 |
| PTPLAD2 | 0.0006231 | 0.0103408 |
| TAOK3 | 0.0006231 | 0.0103408 |
| USP4 | 0.0006231 | 0.0103408 |
| VAMP5 | 0.0006231 | 0.0103408 |
| ZSCAN18 | 0.0006231 | 0.0103408 |
| AFF4 | 0.0007528 | 0.011378 |
| ATF6 | 0.0007528 | 0.011378 |
| C1orf21 | 0.0007528 | 0.011378 |
| CASQ2 | 0.0007528 | 0.011378 |
| CLTB | 0.0007528 | 0.011378 |
| COL15A1 | 0.0007528 | 0.011378 |
| COX7A1 | 0.0007528 | 0.011378 |
| CPPED1 | 0.0007528 | 0.011378 |
| EEA1 | 0.0007528 | 0.011378 |
| EPHA1 | 0.0007528 | 0.011378 |
| FEM1B | 0.0007528 | 0.011378 |
| FRMD4A | 0.0007528 | 0.011378 |
| GRAMD3 | 0.0007528 | 0.011378 |
| HS3ST1 | 0.0007528 | 0.011378 |
| MSRA | 0.0007528 | 0.011378 |
| MTERFD2 | 0.0007528 | 0.011378 |
| PTGER4 | 0.0007528 | 0.011378 |
| RRAS | 0.0007528 | 0.011378 |
| SLMAP | 0.0007528 | 0.011378 |
| SPRR2B | 0.0007528 | 0.011378 |
| STK39 | 0.0007528 | 0.011378 |
| TAGLN | 0.0007528 | 0.011378 |
| TCF7L1 | 0.0007528 | 0.011378 |
| TPSAB1 | 0.0007528 | 0.011378 |
| XPC | 0.0007528 | 0.011378 |
| ZSWIM6 | 0.0007528 | 0.011378 |
| BHLHE40 | 0.0009058 | 0.0124153 |
| BOC | 0.0009058 | 0.0124153 |
| CARD6 | 0.0009058 | 0.0124153 |
| CCT6B | 0.0009058 | 0.0124153 |
| CRNN | 0.0009058 | 0.0124153 |
| CTNNBIP1 | 0.0009058 | 0.0124153 |
| DHRS12 | 0.0009058 | 0.0124153 |
| DUSP22 | 0.0009058 | 0.0124153 |
| ECHDC2 | 0.0009058 | 0.0124153 |
| EPC1 | 0.0009058 | 0.0124153 |
| EPN3 | 0.0009058 | 0.0124153 |
| LYPD3 | 0.0009058 | 0.0124153 |
| MAOA | 0.0009058 | 0.0124153 |
| MYLK | 0.0009058 | 0.0124153 |
| NTN1 | 0.0009058 | 0.0124153 |
| PELI1 | 0.0009058 | 0.0124153 |
| PHYHD1 | 0.0009058 | 0.0124153 |
| PIN4 | 0.0009058 | 0.0124153 |
| PLA2G2A | 0.0009058 | 0.0124153 |
| RAB33B | 0.0009058 | 0.0124153 |
| REEP5 | 0.0009058 | 0.0124153 |
| SMPD1 | 0.0009058 | 0.0124153 |
| TEX2 | 0.0009058 | 0.0124153 |
| TMCC3 | 0.0009058 | 0.0124153 |
| TMEM41B | 0.0009058 | 0.0124153 |
| TRA2A | 0.0009058 | 0.0124153 |
| TRIM22 | 0.0009058 | 0.0124153 |
| UBA7 | 0.0009058 | 0.0124153 |
| ACOX3 | 0.0010844 | 0.0135762 |
| AKAP17A | 0.0010844 | 0.0135762 |
| ANO10 | 0.0010844 | 0.0135762 |
| BTAF1 | 0.0010844 | 0.0135762 |
| CAMSAP1 | 0.0010844 | 0.0135762 |
| FAM13C | 0.0010844 | 0.0135762 |
| FMO2 | 0.0010844 | 0.0135762 |
| FYCO1 | 0.0010844 | 0.0135762 |
| GATSL3 | 0.0010844 | 0.0135762 |
| KLHL20 | 0.0010844 | 0.0135762 |
| MAN1A1 | 0.0010844 | 0.0135762 |
| NCK2 | 0.0010844 | 0.0135762 |
| NDST2 | 0.0010844 | 0.0135762 |
| PDCD4 | 0.0010844 | 0.0135762 |
| RBM5 | 0.0010844 | 0.0135762 |
| RSPO3 | 0.0010844 | 0.0135762 |
| SDPR | 0.0010844 | 0.0135762 |
| SLC24A3 | 0.0010844 | 0.0135762 |
| SLC27A6 | 0.0010844 | 0.0135762 |
| SPINK7 | 0.0010844 | 0.0135762 |
| SYNE1 | 0.0010844 | 0.0135762 |
| TNS1 | 0.0010844 | 0.0135762 |
| TPSB2 | 0.0010844 | 0.0135762 |
| TSPAN4 | 0.0010844 | 0.0135762 |
| ZBTB20 | 0.0010844 | 0.0135762 |
| ZFYVE1 | 0.0010844 | 0.0135762 |
| ACAD8 | 0.0012933 | 0.0147299 |
| AKAP2 | 0.0012933 | 0.0147299 |
| ARHGEF6 | 0.0012933 | 0.0147299 |
| ENDOU | 0.0012933 | 0.0147299 |
| ERCC1 | 0.0012933 | 0.0147299 |
| GPCPD1 | 0.0012933 | 0.0147299 |
| GPSM3 | 0.0012933 | 0.0147299 |
| INSIG1 | 0.0012933 | 0.0147299 |
| IPO8 | 0.0012933 | 0.0147299 |
| LPP | 0.0012933 | 0.0147299 |
| 5-Mar | 0.0012933 | 0.0147299 |
| NDFIP2 | 0.0012933 | 0.0147299 |
| OGN | 0.0012933 | 0.0147299 |
| PALM2-AKAP2 | 0.0012933 | 0.0147299 |
| QPCT | 0.0012933 | 0.0147299 |
| RGS2 | 0.0012933 | 0.0147299 |
| RPS6KA2 | 0.0012933 | 0.0147299 |
| SCAPER | 0.0012933 | 0.0147299 |
| SEPP1 | 0.0012933 | 0.0147299 |
| SERPING1 | 0.0012933 | 0.0147299 |
| SETBP1 | 0.0012933 | 0.0147299 |
| SIAE | 0.0012933 | 0.0147299 |
| SLCO2A1 | 0.0012933 | 0.0147299 |
| SPINK5 | 0.0012933 | 0.0147299 |
| SPRR1B | 0.0012933 | 0.0147299 |
| ST5 | 0.0012933 | 0.0147299 |
| TP53INP2 | 0.0012933 | 0.0147299 |
| TRIM68 | 0.0012933 | 0.0147299 |
| ATP1A2 | 0.0015354 | 0.0161252 |
| CSTA | 0.0015354 | 0.0161252 |
| CYP2C18 | 0.0015354 | 0.0161252 |
| DMXL1 | 0.0015354 | 0.0161252 |
| EFTUD1 | 0.0015354 | 0.0161252 |
| EMCN | 0.0015354 | 0.0161252 |
| ERP44 | 0.0015354 | 0.0161252 |
| HBEGF | 0.0015354 | 0.0161252 |
| KLHL29 | 0.0015354 | 0.0161252 |
| LAMB2 | 0.0015354 | 0.0161252 |
| MALT1 | 0.0015354 | 0.0161252 |
| MTMR6 | 0.0015354 | 0.0161252 |
| MUC5B | 0.0015354 | 0.0161252 |
| PCDHGA9 | 0.0015354 | 0.0161252 |
| PCDHGC5 | 0.0015354 | 0.0161252 |
| PIK3R1 | 0.0015354 | 0.0161252 |
| PROS1 | 0.0015354 | 0.0161252 |
| PRSS22 | 0.0015354 | 0.0161252 |
| PTGDS | 0.0015354 | 0.0161252 |
| PTRF | 0.0015354 | 0.0161252 |
| SPRR1A | 0.0015354 | 0.0161252 |
| SUOX | 0.0015354 | 0.0161252 |
| TBC1D20 | 0.0015354 | 0.0161252 |
| TNRC6B | 0.0015354 | 0.0161252 |
| TRAK2 | 0.0015354 | 0.0161252 |
| TRAPPC8 | 0.0015354 | 0.0161252 |
| UAP1 | 0.0015354 | 0.0161252 |
| UTP14C | 0.0015354 | 0.0161252 |
| C14orf132 | 0.0018165 | 0.0173568 |
| C1orf54 | 0.0018165 | 0.0173568 |
| CLCA2 | 0.0018165 | 0.0173568 |
| CLOCK | 0.0018165 | 0.0173568 |
| CPEB4 | 0.0018165 | 0.0173568 |
| CRYAB | 0.0018165 | 0.0173568 |
| DGKA | 0.0018165 | 0.0173568 |
| EFHC1 | 0.0018165 | 0.0173568 |
| FGL2 | 0.0018165 | 0.0173568 |
| HIPK2 | 0.0018165 | 0.0173568 |
| HLA-DPA1 | 0.0018165 | 0.0173568 |
| HOXB3 | 0.0018165 | 0.0173568 |
| IFI27L2 | 0.0018165 | 0.0173568 |
| LDB2 | 0.0018165 | 0.0173568 |
| LNX2 | 0.0018165 | 0.0173568 |
| LPAR6 | 0.0018165 | 0.0173568 |
| LRRC8A | 0.0018165 | 0.0173568 |
| MAFF | 0.0018165 | 0.0173568 |
| MSRB2 | 0.0018165 | 0.0173568 |
| MYO18A | 0.0018165 | 0.0173568 |
| NAGK | 0.0018165 | 0.0173568 |
| OSBP | 0.0018165 | 0.0173568 |
| PAFAH1B1 | 0.0018165 | 0.0173568 |
| PAQR8 | 0.0018165 | 0.0173568 |
| PEA15 | 0.0018165 | 0.0173568 |
| PECAM1 | 0.0018165 | 0.0173568 |
| PEX14 | 0.0018165 | 0.0173568 |
| PPP1R12B | 0.0018165 | 0.0173568 |
| RBFOX2 | 0.0018165 | 0.0173568 |
| SECISBP2L | 0.0018165 | 0.0173568 |
| SERPINB3 | 0.0018165 | 0.0173568 |
| SGMS1 | 0.0018165 | 0.0173568 |
| SOD3 | 0.0018165 | 0.0173568 |
| ST6GAL1 | 0.0018165 | 0.0173568 |
| TGFBR3 | 0.0018165 | 0.0173568 |
| TMEM43 | 0.0018165 | 0.0173568 |
| TRIP12 | 0.0018165 | 0.0173568 |
| TSPAN5 | 0.0018165 | 0.0173568 |
| UBQLN2 | 0.0018165 | 0.0173568 |
| USO1 | 0.0018165 | 0.0173568 |
| WDR7 | 0.0018165 | 0.0173568 |
| ZBTB45 | 0.0018165 | 0.0173568 |
| ARHGEF3 | 0.0021401 | 0.0189256 |
| BACE2 | 0.0021401 | 0.0189256 |
| CLIC3 | 0.0021401 | 0.0189256 |
| CRCT1 | 0.0021401 | 0.0189256 |
| DENND4C | 0.0021401 | 0.0189256 |
| DPH1 | 0.0021401 | 0.0189256 |
| DSE | 0.0021401 | 0.0189256 |
| ERCC5 | 0.0021401 | 0.0189256 |
| ERO1L | 0.0021401 | 0.0189256 |
| FERMT2 | 0.0021401 | 0.0189256 |
| FOLR1 | 0.0021401 | 0.0189256 |
| GCHFR | 0.0021401 | 0.0189256 |
| GIMAP7 | 0.0021401 | 0.0189256 |
| GNA15 | 0.0021401 | 0.0189256 |
| GPR87 | 0.0021401 | 0.0189256 |
| ITGA2 | 0.0021401 | 0.0189256 |
| KIAA0368 | 0.0021401 | 0.0189256 |
| KRT31 | 0.0021401 | 0.0189256 |
| MACF1 | 0.0021401 | 0.0189256 |
| MFGE8 | 0.0021401 | 0.0189256 |
| MICAL1 | 0.0021401 | 0.0189256 |
| MPZL2 | 0.0021401 | 0.0189256 |
| MUT | 0.0021401 | 0.0189256 |
| NR3C1 | 0.0021401 | 0.0189256 |
| PDLIM3 | 0.0021401 | 0.0189256 |
| RCAN2 | 0.0021401 | 0.0189256 |
| RREB1 | 0.0021401 | 0.0189256 |
| SCML1 | 0.0021401 | 0.0189256 |
| SNRK | 0.0021401 | 0.0189256 |
| SPAG9 | 0.0021401 | 0.0189256 |
| TCEAL3 | 0.0021401 | 0.0189256 |
| TMEM115 | 0.0021401 | 0.0189256 |
| VLDLR | 0.0021401 | 0.0189256 |
| WFDC1 | 0.0021401 | 0.0189256 |
| ZNF211 | 0.0021401 | 0.0189256 |
| ANGPTL1 | 0.0025129 | 0.0207024 |
| ANKRD28 | 0.0025129 | 0.0207024 |
| CAB39 | 0.0025129 | 0.0207024 |
| CCDC53 | 0.0025129 | 0.0207024 |
| DNASE1L3 | 0.0025129 | 0.0207024 |
| DNMBP | 0.0025129 | 0.0207024 |
| EFEMP2 | 0.0025129 | 0.0207024 |
| F2R | 0.0025129 | 0.0207024 |
| FAM129A | 0.0025129 | 0.0207024 |
| FAM179B | 0.0025129 | 0.0207024 |
| GLT8D2 | 0.0025129 | 0.0207024 |
| HIGD1A | 0.0025129 | 0.0207024 |
| HIPK3 | 0.0025129 | 0.0207024 |
| HLA-DPB1 | 0.0025129 | 0.0207024 |
| LATS2 | 0.0025129 | 0.0207024 |
| LNPEP | 0.0025129 | 0.0207024 |
| MOXD1 | 0.0025129 | 0.0207024 |
| MUC1 | 0.0025129 | 0.0207024 |
| PPIC | 0.0025129 | 0.0207024 |
| PPP1R7 | 0.0025129 | 0.0207024 |
| PRDM2 | 0.0025129 | 0.0207024 |
| PTPN13 | 0.0025129 | 0.0207024 |
| QRICH1 | 0.0025129 | 0.0207024 |
| RSBN1 | 0.0025129 | 0.0207024 |
| SNX9 | 0.0025129 | 0.0207024 |
| SPRR3 | 0.0025129 | 0.0207024 |
| STX12 | 0.0025129 | 0.0207024 |
| THSD1 | 0.0025129 | 0.0207024 |
| TOPORS | 0.0025129 | 0.0207024 |
| VPS37B | 0.0025129 | 0.0207024 |
| ZNF25 | 0.0025129 | 0.0207024 |
| ZNF44 | 0.0025129 | 0.0207024 |
| ABCD4 | 0.0029396 | 0.0221725 |
| ABI1 | 0.0029396 | 0.0221725 |
| ABI3BP | 0.0029396 | 0.0221725 |
| AMPD3 | 0.0029396 | 0.0221725 |
| ARL4C | 0.0029396 | 0.0221725 |
| C10orf128 | 0.0029396 | 0.0221725 |
| C3 | 0.0029396 | 0.0221725 |
| CEACAM1 | 0.0029396 | 0.0221725 |
| CHFR | 0.0029396 | 0.0221725 |
| CLDN5 | 0.0029396 | 0.0221725 |
| CNN3 | 0.0029396 | 0.0221725 |
| COL6A2 | 0.0029396 | 0.0221725 |
| CXCL13 | 0.0029396 | 0.0221725 |
| CYP4F3 | 0.0029396 | 0.0221725 |
| DSG3 | 0.0029396 | 0.0221725 |
| DSP | 0.0029396 | 0.0221725 |
| FAM114A2 | 0.0029396 | 0.0221725 |
| GIMAP4 | 0.0029396 | 0.0221725 |
| GMPPB | 0.0029396 | 0.0221725 |
| GNAQ | 0.0029396 | 0.0221725 |
| IL6ST | 0.0029396 | 0.0221725 |
| KCNK6 | 0.0029396 | 0.0221725 |
| LMBR1L | 0.0029396 | 0.0221725 |
| MAP3K4 | 0.0029396 | 0.0221725 |
| MAP3K6 | 0.0029396 | 0.0221725 |
| MBNL1 | 0.0029396 | 0.0221725 |
| MT1H | 0.0029396 | 0.0221725 |
| MYCBP2 | 0.0029396 | 0.0221725 |
| NLRX1 | 0.0029396 | 0.0221725 |
| NNMT | 0.0029396 | 0.0221725 |
| OLFML1 | 0.0029396 | 0.0221725 |
| PRMT2 | 0.0029396 | 0.0221725 |
| RAB2A | 0.0029396 | 0.0221725 |
| RABGAP1L | 0.0029396 | 0.0221725 |
| RABGGTA | 0.0029396 | 0.0221725 |
| RGS1 | 0.0029396 | 0.0221725 |
| RGS5 | 0.0029396 | 0.0221725 |
| SELENBP1 | 0.0029396 | 0.0221725 |
| SMARCA1 | 0.0029396 | 0.0221725 |
| SNX19 | 0.0029396 | 0.0221725 |
| SPRR2C | 0.0029396 | 0.0221725 |
| STEAP4 | 0.0029396 | 0.0221725 |
| STK40 | 0.0029396 | 0.0221725 |
| TBCK | 0.0029396 | 0.0221725 |
| TDRD3 | 0.0029396 | 0.0221725 |
| TMEM214 | 0.0029396 | 0.0221725 |
| TMEM47 | 0.0029396 | 0.0221725 |
| TPCN1 | 0.0029396 | 0.0221725 |
| USP53 | 0.0029396 | 0.0221725 |
| ZNF292 | 0.0029396 | 0.0221725 |
| ZNF337 | 0.0029396 | 0.0221725 |
| ZNF430 | 0.0029396 | 0.0221725 |
| ATAD1 | 0.0034279 | 0.0238422 |
| ATP2B4 | 0.0034279 | 0.0238422 |
| CBL | 0.0034279 | 0.0238422 |
| CD40 | 0.0034279 | 0.0238422 |
| CHKB | 0.0034279 | 0.0238422 |
| CTBS | 0.0034279 | 0.0238422 |
| CTSS | 0.0034279 | 0.0238422 |
| EHBP1 | 0.0034279 | 0.0238422 |
| FAM21A | 0.0034279 | 0.0238422 |
| FAM21B | 0.0034279 | 0.0238422 |
| FAM21C | 0.0034279 | 0.0238422 |
| FAM65A | 0.0034279 | 0.0238422 |
| GALNT1 | 0.0034279 | 0.0238422 |
| GBP3 | 0.0034279 | 0.0238422 |
| GRB7 | 0.0034279 | 0.0238422 |
| HEPH | 0.0034279 | 0.0238422 |
| HOXB2 | 0.0034279 | 0.0238422 |
| IKZF2 | 0.0034279 | 0.0238422 |
| IL6R | 0.0034279 | 0.0238422 |
| KLHL2 | 0.0034279 | 0.0238422 |
| LYNX1 | 0.0034279 | 0.0238422 |
| MAN2A2 | 0.0034279 | 0.0238422 |
| MAP1LC3B | 0.0034279 | 0.0238422 |
| NEAT1 | 0.0034279 | 0.0238422 |
| NECAP2 | 0.0034279 | 0.0238422 |
| NIPAL4 | 0.0034279 | 0.0238422 |
| PACSIN2 | 0.0034279 | 0.0238422 |
| PHC1 | 0.0034279 | 0.0238422 |
| PNPLA8 | 0.0034279 | 0.0238422 |
| PPP2R3A | 0.0034279 | 0.0238422 |
| RASIP1 | 0.0034279 | 0.0238422 |
| RFTN1 | 0.0034279 | 0.0238422 |
| RNF8 | 0.0034279 | 0.0238422 |
| RPRD2 | 0.0034279 | 0.0238422 |
| RRAGC | 0.0034279 | 0.0238422 |
| 8-Sep | 0.0034279 | 0.0238422 |
| SEPW1 | 0.0034279 | 0.0238422 |
| SERPINF1 | 0.0034279 | 0.0238422 |
| SLC7A1 | 0.0034279 | 0.0238422 |
| ST6GALNAC1 | 0.0034279 | 0.0238422 |
| STAB1 | 0.0034279 | 0.0238422 |
| STARD5 | 0.0034279 | 0.0238422 |
| TDRD7 | 0.0034279 | 0.0238422 |
| TECPR1 | 0.0034279 | 0.0238422 |
| TRAK1 | 0.0034279 | 0.0238422 |
| UBR1 | 0.0034279 | 0.0238422 |
| VGLL3 | 0.0034279 | 0.0238422 |
| YIPF6 | 0.0034279 | 0.0238422 |
| ZDHHC17 | 0.0034279 | 0.0238422 |
| ZNF274 | 0.0034279 | 0.0238422 |
| ZNF407 | 0.0034279 | 0.0238422 |
| ALDH7A1 | 0.0039833 | 0.0259142 |
| ARHGAP1 | 0.0039833 | 0.0259142 |
| ATP5G2 | 0.0039833 | 0.0259142 |
| AXL | 0.0039833 | 0.0259142 |
| BAZ2B | 0.0039833 | 0.0259142 |
| C16orf62 | 0.0039833 | 0.0259142 |
| CA12 | 0.0039833 | 0.0259142 |
| CD109 | 0.0039833 | 0.0259142 |
| COL16A1 | 0.0039833 | 0.0259142 |
| CTNNA1 | 0.0039833 | 0.0259142 |
| DECR1 | 0.0039833 | 0.0259142 |
| EFNB2 | 0.0039833 | 0.0259142 |
| EMP3 | 0.0039833 | 0.0259142 |
| FAM135A | 0.0039833 | 0.0259142 |
| FSTL1 | 0.0039833 | 0.0259142 |
| FXYD1 | 0.0039833 | 0.0259142 |
| GABBR1 | 0.0039833 | 0.0259142 |
| GTPBP1 | 0.0039833 | 0.0259142 |
| H2AFJ | 0.0039833 | 0.0259142 |
| HACE1 | 0.0039833 | 0.0259142 |
| HERPUD1 | 0.0039833 | 0.0259142 |
| HTRA1 | 0.0039833 | 0.0259142 |
| IRF7 | 0.0039833 | 0.0259142 |
| ITSN2 | 0.0039833 | 0.0259142 |
| KCND3 | 0.0039833 | 0.0259142 |
| KHDRBS3 | 0.0039833 | 0.0259142 |
| LMO2 | 0.0039833 | 0.0259142 |
| LRBA | 0.0039833 | 0.0259142 |
| MLPH | 0.0039833 | 0.0259142 |
| PARP4 | 0.0039833 | 0.0259142 |
| PCDHGA1 | 0.0039833 | 0.0259142 |
| PDE4C | 0.0039833 | 0.0259142 |
| RBP7 | 0.0039833 | 0.0259142 |
| S100A13 | 0.0039833 | 0.0259142 |
| SASH1 | 0.0039833 | 0.0259142 |
| SATB1 | 0.0039833 | 0.0259142 |
| SCOC | 0.0039833 | 0.0259142 |
| SOX6 | 0.0039833 | 0.0259142 |
| STX17 | 0.0039833 | 0.0259142 |
| SUGP2 | 0.0039833 | 0.0259142 |
| SYNC | 0.0039833 | 0.0259142 |
| TACC1 | 0.0039833 | 0.0259142 |
| TJP2 | 0.0039833 | 0.0259142 |
| TMEM173 | 0.0039833 | 0.0259142 |
| TSPO | 0.0039833 | 0.0259142 |
| URM1 | 0.0039833 | 0.0259142 |
| WDR44 | 0.0039833 | 0.0259142 |
| ZC3H11A | 0.0039833 | 0.0259142 |
| ACADSB | 0.0046148 | 0.0282868 |
| ARRDC3 | 0.0046148 | 0.0282868 |
| BSDC1 | 0.0046148 | 0.0282868 |
| CCBL2 | 0.0046148 | 0.0282868 |
| CRISPLD2 | 0.0046148 | 0.0282868 |
| DNAJB2 | 0.0046148 | 0.0282868 |
| DST | 0.0046148 | 0.0282868 |
| FAM160B1 | 0.0046148 | 0.0282868 |
| FAM8A1 | 0.0046148 | 0.0282868 |
| FBLN5 | 0.0046148 | 0.0282868 |
| FGFBP1 | 0.0046148 | 0.0282868 |
| FRMD6 | 0.0046148 | 0.0282868 |
| IL1R1 | 0.0046148 | 0.0282868 |
| ITPKC | 0.0046148 | 0.0282868 |
| ITPR1 | 0.0046148 | 0.0282868 |
| KRT23 | 0.0046148 | 0.0282868 |
| LYSMD3 | 0.0046148 | 0.0282868 |
| MAP1LC3A | 0.0046148 | 0.0282868 |
| MYO9A | 0.0046148 | 0.0282868 |
| NFIA | 0.0046148 | 0.0282868 |
| NME7 | 0.0046148 | 0.0282868 |
| NOTCH2 | 0.0046148 | 0.0282868 |
| NUAK2 | 0.0046148 | 0.0282868 |
| PCMTD1 | 0.0046148 | 0.0282868 |
| PJA2 | 0.0046148 | 0.0282868 |
| PLS3 | 0.0046148 | 0.0282868 |
| POLR3GL | 0.0046148 | 0.0282868 |
| PYROXD1 | 0.0046148 | 0.0282868 |
| RGL2 | 0.0046148 | 0.0282868 |
| RPS6KA3 | 0.0046148 | 0.0282868 |
| SDC1 | 0.0046148 | 0.0282868 |
| SKI | 0.0046148 | 0.0282868 |
| SLC15A3 | 0.0046148 | 0.0282868 |
| SLC31A2 | 0.0046148 | 0.0282868 |
| SUV420H1 | 0.0046148 | 0.0282868 |
| SVIL | 0.0046148 | 0.0282868 |
| THYN1 | 0.0046148 | 0.0282868 |
| TPM2 | 0.0046148 | 0.0282868 |
| TXNDC15 | 0.0046148 | 0.0282868 |
| UIMC1 | 0.0046148 | 0.0282868 |
| VPS13D | 0.0046148 | 0.0282868 |
| YPEL5 | 0.0046148 | 0.0282868 |
| AGTPBP1 | 0.0053292 | 0.0304583 |
| ARAP2 | 0.0053292 | 0.0304583 |
| ARHGAP6 | 0.0053292 | 0.0304583 |
| BCAR3 | 0.0053292 | 0.0304583 |
| BTG2 | 0.0053292 | 0.0304583 |
| C18orf32 | 0.0053292 | 0.0304583 |
| C1orf116 | 0.0053292 | 0.0304583 |
| C9orf72 | 0.0053292 | 0.0304583 |
| CAPN7 | 0.0053292 | 0.0304583 |
| CCDC71L | 0.0053292 | 0.0304583 |
| CHD7 | 0.0053292 | 0.0304583 |
| CLN5 | 0.0053292 | 0.0304583 |
| COG5 | 0.0053292 | 0.0304583 |
| CRIP2 | 0.0053292 | 0.0304583 |
| DPYSL2 | 0.0053292 | 0.0304583 |
| EIF2AK3 | 0.0053292 | 0.0304583 |
| FHOD3 | 0.0053292 | 0.0304583 |
| GATM | 0.0053292 | 0.0304583 |
| GNAI2 | 0.0053292 | 0.0304583 |
| GNPAT | 0.0053292 | 0.0304583 |
| GOLGB1 | 0.0053292 | 0.0304583 |
| GYS1 | 0.0053292 | 0.0304583 |
| HINFP | 0.0053292 | 0.0304583 |
| HIST1H2BG | 0.0053292 | 0.0304583 |
| JUND | 0.0053292 | 0.0304583 |
| KDM4B | 0.0053292 | 0.0304583 |
| KIAA0040 | 0.0053292 | 0.0304583 |
| KIAA0355 | 0.0053292 | 0.0304583 |
| LMAN1 | 0.0053292 | 0.0304583 |
| MAF | 0.0053292 | 0.0304583 |
| MAP3K14 | 0.0053292 | 0.0304583 |
| MCC | 0.0053292 | 0.0304583 |
| MGP | 0.0053292 | 0.0304583 |
| MOSPD1 | 0.0053292 | 0.0304583 |
| NAA16 | 0.0053292 | 0.0304583 |
| NDST1 | 0.0053292 | 0.0304583 |
| NUPR1 | 0.0053292 | 0.0304583 |
| ORAI3 | 0.0053292 | 0.0304583 |
| PAPSS1 | 0.0053292 | 0.0304583 |
| PDIK1L | 0.0053292 | 0.0304583 |
| PRSS27 | 0.0053292 | 0.0304583 |
| RAP1GDS1 | 0.0053292 | 0.0304583 |
| RNF38 | 0.0053292 | 0.0304583 |
| S1PR3 | 0.0053292 | 0.0304583 |
| SACS | 0.0053292 | 0.0304583 |
| SCNN1G | 0.0053292 | 0.0304583 |
| SRRM1 | 0.0053292 | 0.0304583 |
| SULT2B1 | 0.0053292 | 0.0304583 |
| THBS3 | 0.0053292 | 0.0304583 |
| TIPARP | 0.0053292 | 0.0304583 |
| TMEM120A | 0.0053292 | 0.0304583 |
| TMEM161B | 0.0053292 | 0.0304583 |
| TPPP3 | 0.0053292 | 0.0304583 |
| TTC37 | 0.0053292 | 0.0304583 |
| USP6NL | 0.0053292 | 0.0304583 |
| VPS13A | 0.0053292 | 0.0304583 |
| XK | 0.0053292 | 0.0304583 |
| ZNF426 | 0.0053292 | 0.0304583 |
| ABCA1 | 0.0061368 | 0.0330538 |
| ACADM | 0.0061368 | 0.0330538 |
| ADAMTS5 | 0.0061368 | 0.0330538 |
| ARGLU1 | 0.0061368 | 0.0330538 |
| BTBD7 | 0.0061368 | 0.0330538 |
| CDH13 | 0.0061368 | 0.0330538 |
| CH25H | 0.0061368 | 0.0330538 |
| CLU | 0.0061368 | 0.0330538 |
| CXCR2 | 0.0061368 | 0.0330538 |
| CYP51A1 | 0.0061368 | 0.0330538 |
| DPAGT1 | 0.0061368 | 0.0330538 |
| DUSP18 | 0.0061368 | 0.0330538 |
| ENG | 0.0061368 | 0.0330538 |
| EPS8L1 | 0.0061368 | 0.0330538 |
| FMOD | 0.0061368 | 0.0330538 |
| GABRP | 0.0061368 | 0.0330538 |
| GOLGA2 | 0.0061368 | 0.0330538 |
| GPR116 | 0.0061368 | 0.0330538 |
| GSK3A | 0.0061368 | 0.0330538 |
| HECW2 | 0.0061368 | 0.0330538 |
| HLA-DMB | 0.0061368 | 0.0330538 |
| HSPG2 | 0.0061368 | 0.0330538 |
| KAT5 | 0.0061368 | 0.0330538 |
| KIT | 0.0061368 | 0.0330538 |
| MAP3K3 | 0.0061368 | 0.0330538 |
| NBEA | 0.0061368 | 0.0330538 |
| NBL1 | 0.0061368 | 0.0330538 |
| NUCB2 | 0.0061368 | 0.0330538 |
| OAS1 | 0.0061368 | 0.0330538 |
| PBXIP1 | 0.0061368 | 0.0330538 |
| PDLIM2 | 0.0061368 | 0.0330538 |
| PERP | 0.0061368 | 0.0330538 |
| PLXDC2 | 0.0061368 | 0.0330538 |
| PODN | 0.0061368 | 0.0330538 |
| PRKAR2B | 0.0061368 | 0.0330538 |
| RCBTB2 | 0.0061368 | 0.0330538 |
| RGS14 | 0.0061368 | 0.0330538 |
| RNF150 | 0.0061368 | 0.0330538 |
| ROBO1 | 0.0061368 | 0.0330538 |
| ROCK1 | 0.0061368 | 0.0330538 |
| RPL27A | 0.0061368 | 0.0330538 |
| SCNN1B | 0.0061368 | 0.0330538 |
| SEMA3F | 0.0061368 | 0.0330538 |
| SIDT2 | 0.0061368 | 0.0330538 |
| SLC2A10 | 0.0061368 | 0.0330538 |
| SPTLC1 | 0.0061368 | 0.0330538 |
| STAT6 | 0.0061368 | 0.0330538 |
| TACC2 | 0.0061368 | 0.0330538 |
| TBC1D17 | 0.0061368 | 0.0330538 |
| TMEM159 | 0.0061368 | 0.0330538 |
| TRIM39 | 0.0061368 | 0.0330538 |
| VWF | 0.0061368 | 0.0330538 |
| YPEL3 | 0.0061368 | 0.0330538 |
| ZDHHC3 | 0.0061368 | 0.0330538 |
| ZYG11B | 0.0061368 | 0.0330538 |
| ACSS2 | 0.0070452 | 0.0361116 |
| AKAP12 | 0.0070452 | 0.0361116 |
| ANKRD49 | 0.0070452 | 0.0361116 |
| APP | 0.0070452 | 0.0361116 |
| ARHGAP15 | 0.0070452 | 0.0361116 |
| BMP2 | 0.0070452 | 0.0361116 |
| CGRRF1 | 0.0070452 | 0.0361116 |
| COL17A1 | 0.0070452 | 0.0361116 |
| CYB5R2 | 0.0070452 | 0.0361116 |
| DNAJC3 | 0.0070452 | 0.0361116 |
| DPT | 0.0070452 | 0.0361116 |
| DYNLT3 | 0.0070452 | 0.0361116 |
| DYRK1A | 0.0070452 | 0.0361116 |
| FAM134A | 0.0070452 | 0.0361116 |
| FAM63A | 0.0070452 | 0.0361116 |
| FBLN2 | 0.0070452 | 0.0361116 |
| FEM1C | 0.0070452 | 0.0361116 |
| FKBP1A | 0.0070452 | 0.0361116 |
| FOXN3 | 0.0070452 | 0.0361116 |
| GPR183 | 0.0070452 | 0.0361116 |
| GRAMD4 | 0.0070452 | 0.0361116 |
| GTF2A1 | 0.0070452 | 0.0361116 |
| GTPBP2 | 0.0070452 | 0.0361116 |
| GULP1 | 0.0070452 | 0.0361116 |
| HOOK3 | 0.0070452 | 0.0361116 |
| KIF1B | 0.0070452 | 0.0361116 |
| MAL2 | 0.0070452 | 0.0361116 |
| MYADM | 0.0070452 | 0.0361116 |
| MYH9 | 0.0070452 | 0.0361116 |
| NAV1 | 0.0070452 | 0.0361116 |
| OGT | 0.0070452 | 0.0361116 |
| PAMR1 | 0.0070452 | 0.0361116 |
| PCBP4 | 0.0070452 | 0.0361116 |
| PCDHGB7 | 0.0070452 | 0.0361116 |
| PHC2 | 0.0070452 | 0.0361116 |
| PHLDA2 | 0.0070452 | 0.0361116 |
| RAB9A | 0.0070452 | 0.0361116 |
| SERINC5 | 0.0070452 | 0.0361116 |
| SH3GLB1 | 0.0070452 | 0.0361116 |
| SREK1IP1 | 0.0070452 | 0.0361116 |
| SRGN | 0.0070452 | 0.0361116 |
| STAMBP | 0.0070452 | 0.0361116 |
| SYNPO2 | 0.0070452 | 0.0361116 |
| TFB1M | 0.0070452 | 0.0361116 |
| TJP1 | 0.0070452 | 0.0361116 |
| TMX4 | 0.0070452 | 0.0361116 |
| TOB1 | 0.0070452 | 0.0361116 |
| UBE2J1 | 0.0070452 | 0.0361116 |
| ZBTB5 | 0.0070452 | 0.0361116 |
| ZNF423 | 0.0070452 | 0.0361116 |
| ZRSR2 | 0.0070452 | 0.0361116 |
| ADCY9 | 0.0080663 | 0.0389832 |
| AKAP9 | 0.0080663 | 0.0389832 |
| ALOX15B | 0.0080663 | 0.0389832 |
| AP3S1 | 0.0080663 | 0.0389832 |
| APPBP2 | 0.0080663 | 0.0389832 |
| AQP3 | 0.0080663 | 0.0389832 |
| BARD1 | 0.0080663 | 0.0389832 |
| C2CD2 | 0.0080663 | 0.0389832 |
| C2orf54 | 0.0080663 | 0.0389832 |
| CBLL1 | 0.0080663 | 0.0389832 |
| CD74 | 0.0080663 | 0.0389832 |
| CD93 | 0.0080663 | 0.0389832 |
| DDIT4 | 0.0080663 | 0.0389832 |
| DMTF1 | 0.0080663 | 0.0389832 |
| DOCK10 | 0.0080663 | 0.0389832 |
| DOCK8 | 0.0080663 | 0.0389832 |
| ETS2 | 0.0080663 | 0.0389832 |
| FOSB | 0.0080663 | 0.0389832 |
| GLRX | 0.0080663 | 0.0389832 |
| GLT8D1 | 0.0080663 | 0.0389832 |
| GNA13 | 0.0080663 | 0.0389832 |
| HIST2H2BE | 0.0080663 | 0.0389832 |
| HLA-E | 0.0080663 | 0.0389832 |
| LEMD3 | 0.0080663 | 0.0389832 |
| MAN2B2 | 0.0080663 | 0.0389832 |
| MAST2 | 0.0080663 | 0.0389832 |
| MID1IP1 | 0.0080663 | 0.0389832 |
| MYL12A | 0.0080663 | 0.0389832 |
| NBEAL2 | 0.0080663 | 0.0389832 |
| NDUFA11 | 0.0080663 | 0.0389832 |
| NFIB | 0.0080663 | 0.0389832 |
| NPC1 | 0.0080663 | 0.0389832 |
| OFD1 | 0.0080663 | 0.0389832 |
| PCDHGA4 | 0.0080663 | 0.0389832 |
| PPIL3 | 0.0080663 | 0.0389832 |
| PPP3CA | 0.0080663 | 0.0389832 |
| PRKG1 | 0.0080663 | 0.0389832 |
| PVRL1 | 0.0080663 | 0.0389832 |
| RAB11FIP2 | 0.0080663 | 0.0389832 |
| RAB24 | 0.0080663 | 0.0389832 |
| RAB8B | 0.0080663 | 0.0389832 |
| S100A4 | 0.0080663 | 0.0389832 |
| SCEL | 0.0080663 | 0.0389832 |
| SEC63 | 0.0080663 | 0.0389832 |
| SERPINB1 | 0.0080663 | 0.0389832 |
| SFTA2 | 0.0080663 | 0.0389832 |
| SH3KBP1 | 0.0080663 | 0.0389832 |
| SLC19A2 | 0.0080663 | 0.0389832 |
| SPTLC2 | 0.0080663 | 0.0389832 |
| STX7 | 0.0080663 | 0.0389832 |
| STYK1 | 0.0080663 | 0.0389832 |
| TMEM63B | 0.0080663 | 0.0389832 |
| TMOD3 | 0.0080663 | 0.0389832 |
| TUBA4A | 0.0080663 | 0.0389832 |
| VAMP8 | 0.0080663 | 0.0389832 |
| WASF3 | 0.0080663 | 0.0389832 |
| ZFPM2 | 0.0080663 | 0.0389832 |
| ZNF264 | 0.0080663 | 0.0389832 |
| AACS | 0.0092091 | 0.0415037 |
| AEBP1 | 0.0092091 | 0.0415037 |
| ANKRD12 | 0.0092091 | 0.0415037 |
| ARMCX3 | 0.0092091 | 0.0415037 |
| AUH | 0.0092091 | 0.0415037 |
| BOK | 0.0092091 | 0.0415037 |
| C19orf66 | 0.0092091 | 0.0415037 |
| CAP1 | 0.0092091 | 0.0415037 |
| CCL11 | 0.0092091 | 0.0415037 |
| CCNDBP1 | 0.0092091 | 0.0415037 |
| CLK1 | 0.0092091 | 0.0415037 |
| CREB3L2 | 0.0092091 | 0.0415037 |
| CRYZ | 0.0092091 | 0.0415037 |
| CTSO | 0.0092091 | 0.0415037 |
| DICER1 | 0.0092091 | 0.0415037 |
| DSC3 | 0.0092091 | 0.0415037 |
| DVL1 | 0.0092091 | 0.0415037 |
| EDN1 | 0.0092091 | 0.0415037 |
| FASN | 0.0092091 | 0.0415037 |
| FOLR2 | 0.0092091 | 0.0415037 |
| FRMD8 | 0.0092091 | 0.0415037 |
| FZD10 | 0.0092091 | 0.0415037 |
| GHR | 0.0092091 | 0.0415037 |
| GOLGA8B | 0.0092091 | 0.0415037 |
| GPR75 | 0.0092091 | 0.0415037 |
| HKR1 | 0.0092091 | 0.0415037 |
| HLA-DMA | 0.0092091 | 0.0415037 |
| HMCN1 | 0.0092091 | 0.0415037 |
| ING2 | 0.0092091 | 0.0415037 |
| JAG1 | 0.0092091 | 0.0415037 |
| KMT2B | 0.0092091 | 0.0415037 |
| LDOC1 | 0.0092091 | 0.0415037 |
| LIG4 | 0.0092091 | 0.0415037 |
| LY75 | 0.0092091 | 0.0415037 |
| MFSD1 | 0.0092091 | 0.0415037 |
| MX1 | 0.0092091 | 0.0415037 |
| MYO5A | 0.0092091 | 0.0415037 |
| NDUFB3 | 0.0092091 | 0.0415037 |
| PLOD2 | 0.0092091 | 0.0415037 |
| PNRC1 | 0.0092091 | 0.0415037 |
| PPP2CB | 0.0092091 | 0.0415037 |
| PPP4R1 | 0.0092091 | 0.0415037 |
| PRSS8 | 0.0092091 | 0.0415037 |
| PTCRA | 0.0092091 | 0.0415037 |
| RAD23B | 0.0092091 | 0.0415037 |
| RALA | 0.0092091 | 0.0415037 |
| RAP1A | 0.0092091 | 0.0415037 |
| SEC31B | 0.0092091 | 0.0415037 |
| SESTD1 | 0.0092091 | 0.0415037 |
| SLAMF7 | 0.0092091 | 0.0415037 |
| SLC1A1 | 0.0092091 | 0.0415037 |
| SLC26A2 | 0.0092091 | 0.0415037 |
| SLC35A1 | 0.0092091 | 0.0415037 |
| SOX15 | 0.0092091 | 0.0415037 |
| STAM2 | 0.0092091 | 0.0415037 |
| STK36 | 0.0092091 | 0.0415037 |
| TCEAL4 | 0.0092091 | 0.0415037 |
| TCTA | 0.0092091 | 0.0415037 |
| TEAD1 | 0.0092091 | 0.0415037 |
| TMEM158 | 0.0092091 | 0.0415037 |
| TMEM176A | 0.0092091 | 0.0415037 |
| TMEM57 | 0.0092091 | 0.0415037 |
| TOLLIP | 0.0092091 | 0.0415037 |
| TP53INP1 | 0.0092091 | 0.0415037 |
| TRIM44 | 0.0092091 | 0.0415037 |
| TRPS1 | 0.0092091 | 0.0415037 |
| WASL | 0.0092091 | 0.0415037 |
| ZNF93 | 0.0092091 | 0.0415037 |
| ACOX1 | 0.0104869 | 0.0448231 |
| AIF1 | 0.0104869 | 0.0448231 |
| AP1S2 | 0.0104869 | 0.0448231 |
| APOLD1 | 0.0104869 | 0.0448231 |
| ARHGEF18 | 0.0104869 | 0.0448231 |
| ATP6V1D | 0.0104869 | 0.0448231 |
| ATP7A | 0.0104869 | 0.0448231 |
| CAPZA2 | 0.0104869 | 0.0448231 |
| CIDEB | 0.0104869 | 0.0448231 |
| CTNNAL1 | 0.0104869 | 0.0448231 |
| DDX60 | 0.0104869 | 0.0448231 |
| EPB41L4A | 0.0104869 | 0.0448231 |
| EPHX3 | 0.0104869 | 0.0448231 |
| FAM117A | 0.0104869 | 0.0448231 |
| FOXN2 | 0.0104869 | 0.0448231 |
| GABPA | 0.0104869 | 0.0448231 |
| ITIH5 | 0.0104869 | 0.0448231 |
| ITPR2 | 0.0104869 | 0.0448231 |
| JUN | 0.0104869 | 0.0448231 |
| JUP | 0.0104869 | 0.0448231 |
| LPAR5 | 0.0104869 | 0.0448231 |
| LUM | 0.0104869 | 0.0448231 |
| LYZ | 0.0104869 | 0.0448231 |
| MAPK7 | 0.0104869 | 0.0448231 |
| MARK4 | 0.0104869 | 0.0448231 |
| MMP2 | 0.0104869 | 0.0448231 |
| MSL3 | 0.0104869 | 0.0448231 |
| MVP | 0.0104869 | 0.0448231 |
| NAAA | 0.0104869 | 0.0448231 |
| NRIP1 | 0.0104869 | 0.0448231 |
| PCDHGA2 | 0.0104869 | 0.0448231 |
| PCF11 | 0.0104869 | 0.0448231 |
| PDZD8 | 0.0104869 | 0.0448231 |
| PLA2G4C | 0.0104869 | 0.0448231 |
| PTGFRN | 0.0104869 | 0.0448231 |
| PTTG1IP | 0.0104869 | 0.0448231 |
| RAB11FIP5 | 0.0104869 | 0.0448231 |
| REEP6 | 0.0104869 | 0.0448231 |
| RHOB | 0.0104869 | 0.0448231 |
| RIOK3 | 0.0104869 | 0.0448231 |
| RSU1 | 0.0104869 | 0.0448231 |
| S100A9 | 0.0104869 | 0.0448231 |
| SERINC1 | 0.0104869 | 0.0448231 |
| SLC44A2 | 0.0104869 | 0.0448231 |
| SRI | 0.0104869 | 0.0448231 |
| TCEA3 | 0.0104869 | 0.0448231 |
| TFAP2A | 0.0104869 | 0.0448231 |
| TGFBR2 | 0.0104869 | 0.0448231 |
| UBE2D3 | 0.0104869 | 0.0448231 |
| UXT | 0.0104869 | 0.0448231 |
| VIM | 0.0104869 | 0.0448231 |
| WDR48 | 0.0104869 | 0.0448231 |
| ZC3H7A | 0.0104869 | 0.0448231 |
| ZKSCAN1 | 0.0104869 | 0.0448231 |
| ZYX | 0.0104869 | 0.0448231 |
| AIDA | 0.0119098 | 0.0484313 |
| APOBEC3C | 0.0119098 | 0.0484313 |
| ARHGEF5 | 0.0119098 | 0.0484313 |
| BCL2 | 0.0119098 | 0.0484313 |
| BLVRA | 0.0119098 | 0.0484313 |
| BTBD10 | 0.0119098 | 0.0484313 |
| C4A | 0.0119098 | 0.0484313 |
| CCDC3 | 0.0119098 | 0.0484313 |
| CEACAM5 | 0.0119098 | 0.0484313 |
| CHMP5 | 0.0119098 | 0.0484313 |
| DAB2 | 0.0119098 | 0.0484313 |
| DLL1 | 0.0119098 | 0.0484313 |
| DNAJB9 | 0.0119098 | 0.0484313 |
| ETFDH | 0.0119098 | 0.0484313 |
| FOXF1 | 0.0119098 | 0.0484313 |
| GBE1 | 0.0119098 | 0.0484313 |
| GBP2 | 0.0119098 | 0.0484313 |
| GLG1 | 0.0119098 | 0.0484313 |
| GOLPH3L | 0.0119098 | 0.0484313 |
| IBTK | 0.0119098 | 0.0484313 |
| IFI27 | 0.0119098 | 0.0484313 |
| JMJD6 | 0.0119098 | 0.0484313 |
| KIAA0141 | 0.0119098 | 0.0484313 |
| KIF5B | 0.0119098 | 0.0484313 |
| KLF10 | 0.0119098 | 0.0484313 |
| KRCC1 | 0.0119098 | 0.0484313 |
| LAD1 | 0.0119098 | 0.0484313 |
| LONRF1 | 0.0119098 | 0.0484313 |
| MAP7D1 | 0.0119098 | 0.0484313 |
| MSH3 | 0.0119098 | 0.0484313 |
| MYH11 | 0.0119098 | 0.0484313 |
| NDEL1 | 0.0119098 | 0.0484313 |
| NFIC | 0.0119098 | 0.0484313 |
| NUBP1 | 0.0119098 | 0.0484313 |
| PALLD | 0.0119098 | 0.0484313 |
| PPIP5K2 | 0.0119098 | 0.0484313 |
| RAB6A | 0.0119098 | 0.0484313 |
| RANBP9 | 0.0119098 | 0.0484313 |
| RASSF7 | 0.0119098 | 0.0484313 |
| RGS3 | 0.0119098 | 0.0484313 |
| RPGR | 0.0119098 | 0.0484313 |
| SEMA4B | 0.0119098 | 0.0484313 |
| SETD2 | 0.0119098 | 0.0484313 |
| SLC25A23 | 0.0119098 | 0.0484313 |
| SLC25A44 | 0.0119098 | 0.0484313 |
| SLPI | 0.0119098 | 0.0484313 |
| SP1 | 0.0119098 | 0.0484313 |
| SPAG7 | 0.0119098 | 0.0484313 |
| THBD | 0.0119098 | 0.0484313 |
| TMEM167A | 0.0119098 | 0.0484313 |
| TNIP1 | 0.0119098 | 0.0484313 |
| TPBG | 0.0119098 | 0.0484313 |
| TPRG1L | 0.0119098 | 0.0484313 |
| TRIM13 | 0.0119098 | 0.0484313 |
| UBP1 | 0.0119098 | 0.0484313 |
| UBR2 | 0.0119098 | 0.0484313 |
| ZZEF1 | 0.0119098 | 0.0484313 |

EC: esophageal carcinoma; FDR: false discovery rate.

**Table S2** **Basic information of 5 EC patients for qRT-PCR validation**

| **Patient No.** | **Age** | **Gender** | **Part of thoracic** | **Histological type** | **TNM staging** |
| --- | --- | --- | --- | --- | --- |
| 1 | 69 | male | upper | ESCC | T3N0M0 |
| 2 | 71 | male | middle | ESCC | T3N2M0 |
| 3 | 61 | male | middle | ESCC | T3N0M0 |
| 4 | 48 | female | middle | ESCC | T3N1M0 |
| 5 | 62 | male | upper | ESCC | T3N0M0 |

No.: number; EC; esophageal carcinoma; ESCC: esophageal squamous cell carcinoma; TNM: tumor, node and metastasis; qRT-PCR: quantitative real-time polymerase chain reaction.

**Table S3 Genes and primers of qRT-PCR**

| **Genes** | **Primer sequence (5'to3')** |
| --- | --- |
| CCT3 | Forward- CCAGCACCATCCGTCTACTTACCT |
|  | Reverse- CCGTCTCACCATTTACACCCCAG |
| MYBL2 | Forward- CCTCCGTCCCTCCTACCATAA |
|  | Reverse- CACTTGTAAGGCAGGCTCGTT |
| CDK4 | Forward- GAGCATCCCAATGTTGTCCG |
|  | Reverse- GAAACTGGCGCATCAGATCC |
| CENPF | Forward- GTAAAGAAAGGGTTTGCTGACATC |
|  | Reverse- CAGGAAGATTATTGACTGGGACG |
| CDKN3 | Forward- ATCACCCATCATCATCCAATCG |
|  | Reverse- TCTCCCAAGTCCTCCATAGCAG |
| CDCA3 | Forward- CCTATGAAGACCAGCAGTGGAGAC |
|  | Reverse- GGTTTCTGTGGGCTGTCTTGC |
| SIM2 | Forward- ACCCAAAAATACCAAGATGAAGAC |
|  | Reverse- AGGCTGTAGGATGGCGTGTA |
| THSD4 | Forward- CGGGCAAGGTGGAGTGGTT |
|  | Reverse- AGGCTTGAGGTGGCAGGATT |
| β-actin | Forward- CTGAAGTACCCCATCGAGCAC |
|  | Reverse- ATAGCACAGCCTGGATAGCAAC |

qRT-PCR: Quantitative real-time polymerase chain reaction.
